# Diversity in Chemical Structures and Biological Properties of Plant Alkaloids

**DOI:** 10.3390/molecules26113374

**Published:** 2021-06-03

**Authors:** Sweta Bhambhani, Kirtikumar R. Kondhare, Ashok P. Giri

**Affiliations:** 1 CSIR-National Chemical Laboratory, Plant Molecular Biology Unit, Biochemical Sciences Division, Dr. Homi Bhabha Road, Pune 411008, Maharashtra, India; s.bhambhani@ncl.res.in (S.B.); kr.kondhare@ncl.res.in (K.R.K.); 2 Academy of Scientific and Innovative Research (AcSIR), Ghaziabad 201002, Uttar Pradesh, India

**Keywords:** alkaloid, biological activity, classification, modification, enzyme, defense

## Abstract

Phytochemicals belonging to the group of alkaloids are signature specialized metabolites endowed with countless biological activities. Plants are armored with these naturally produced nitrogenous compounds to combat numerous challenging environmental stress conditions. Traditional and modern healthcare systems have harnessed the potential of these organic compounds for the treatment of many ailments. Various chemical entities (functional groups) attached to the central moiety are responsible for their diverse range of biological properties. The development of the characterization of these plant metabolites and the enzymes involved in their biosynthesis is of an utmost priority to deliver enhanced advantages in terms of biological properties and productivity. Further, the incorporation of whole/partial metabolic pathways in the heterologous system and/or the overexpression of biosynthetic steps in homologous systems have both become alternative and lucrative methods over chemical synthesis in recent times. Moreover, in-depth research on alkaloid biosynthetic pathways has revealed numerous chemical modifications that occur during alkaloidal conversions. These chemical reactions involve glycosylation, acylation, reduction, oxidation, and methylation steps, and they are usually responsible for conferring the biological activities possessed by alkaloids. In this review, we aim to discuss the alkaloidal group of plant specialized metabolites and their brief classification covering major categories. We also emphasize the diversity in the basic structures of plant alkaloids arising through enzymatically catalyzed structural modifications in certain plant species, as well as their emerging diverse biological activities. The role of alkaloids in plant defense and their mechanisms of action are also briefly discussed. Moreover, the commercial utilization of plant alkaloids in the marketplace displaying various applications has been enumerated.

## 1. Introduction

Plants possess a diverse array of metabolic products arising from both primary and secondary metabolisms. Primary metabolites exist in every living cell produced from vital metabolic reactions. Conversely, specialized metabolites are derived from the primary metabolism and are present only in prominent tissues required for specific functions [1]. Alkaloids are naturally occurring specialized metabolites with nitrogen as a characteristic element present in their chemical structures. The treasure of the biological potency of alkaloids is attributed to the different arrangement of the atoms within their chemical structures.

Across the kingdoms, alkaloids occur with different chemical structures and attached functional entities, displaying wide-reaching biological properties. Organisms from the marine world such as shellfish and sponges contain alkaloids such as pinnatoxins, pinnamine, and halochlorine, which are found to be useful for treating cardiac and non-cardiac inflammatory ailments [2]. Alkaloids act as defensive chemicals in many ladybird beetles, which secrete hemolymph-containing bitter alkaloids upon molestation. Few ant species have alkaloids like *cis-* and *trans*-2-methyl-6-alkylpiperidines along with proteinaceous substances in their poisonous venom, which are used for both defensive and offensive purposes [3].

Traditionally, plant extracts have been used as medicines in healthcare systems. Since the nineteenth century, the bioactivity of these compounds has been utilized for the production of therapeutic and psychoactive drugs [4]. Recently, the compounds synthesized in different plant tissues have been extensively studied for their biosynthesis and biological activities [5]. Pharmaceutical industries have utilized these naturally occurring compounds to develop formulations for better therapeutic potentials. These alkaloids show activities ranging from medicinal to acute toxicity, such as in the case of poppy alkaloids, depending upon the dosage of compounds [6]. Higher plant species belonging to the Berberidaceae, Amaryllidaceae, Liliaceae, Leguminaceae, Papaveraceae, Ranunculaceae, and Solanaceae families are prominently rich in alkaloids, based on reports to date [7]. Furthermore, different classes of alkaloids are found across different families, which in turn depends on the active biosynthetic pathway in a particular species. The detailed study of specialized metabolite biosynthesis through chemical and biotechnological approaches has created a comprehensive understanding of the diversity in alkaloids and their precursors.

In this review, firstly, we describe the classification of alkaloids according to different aspects. Subsequently, five major and crucial chemical transformations to the backbone of alkaloids, such as glycosylation, acylation, reduction, oxidation, and methylation, have been discussed along with the diversified biological activities of alkaloids. The role of alkaloids in plant defense and their commercial importance have also been described in the latter sections.

## 2. Classification of Plant Alkaloids

**Figure 1:**
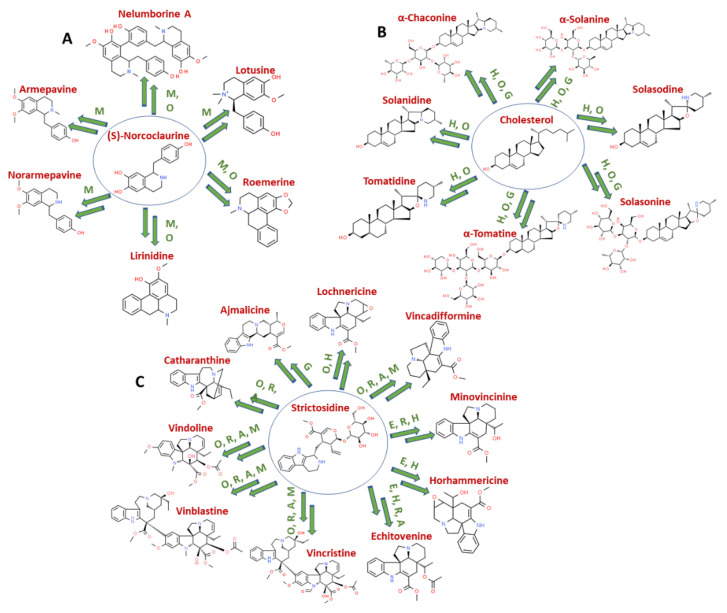
Examples of alkaloids biosynthesized from the common skeleton. Multiple alkaloids are biosynthesized from the common skeleton represented inside the blue circle in different plant species. (**A**) Benzylisoquinoline alkaloids in Sacred lotus; (**B**) Steroidal alkaloids and their glycosides in tomato, potato, and eggplant; (**C**) Terpene indole alkaloids in *C. roseus*. The common skeleton undergoes multiple enzymatic conversions (M = methylation, O = oxidation, R = reduction, G = glycosylation, A = acetylation, H = hydroxylation, and E = epoxidation) represented by multiple arrows to form a variety of alkaloids. Key enzymatic reactions that are reported have been mentioned beside the arrows. The chemical structures of alkaloids are drawn from “ChemSpider: the Free Chemical Database”.

The arrangement and combination of functional groups result in the production of a diverse range of alkaloids in both the plant and animal kingdoms. This broad class of specialized alkaloids has been further classified according to different aspects in plants, such as their biosynthesis pathways, chemical structures, and taxonomical groups [8] (Table 1). Many alkaloids share a common skeleton within a particular genus of plants, however differ in their chemical and biological properties. The important groups of such alkaloids are depicted in Figure 1.

### 2.1. Biosynthetic Pathway

In this category, alkaloids are grouped on the criteria of being biosynthesized from the same/similar biochemical precursors, which after going through certain chemical reactions gives rise to stable alkaloids (Table 1). Tyrosine, tryptophan, ornithine, and lysine are amino acid precursors, which undergo enzymatically catalyzed chemical reactions giving rise to tetrahydroisoquinoline, indole, pyrrolizidine, and piperidine alkaloids, respectively [42]. For example, L-aspartate acts as a precursor for the biosynthesis of pyridine- and pyridinone-type alkaloids. Nicotinic alkaloids, such as nicotine and anabasine, are pyridine-type alkaloids containing nicotinic acid in their partial structure, whereas cerpegin is a pyridinone-type alkaloid, whose biosynthetic route is similar to the nicotine biosynthetic pathway [18,19]. Some non-amino acid compounds also act as precursors by supplying a nitrogen atom for alkaloid biosynthesis, such as anthranillic acid, which gives rise to quinoline and quinazoline alkaloids. Xanthine alkaloids include caffeine and theobromine, derived from a nucleoside, adenosine. Steroid and terpenoid alkaloids are pseudoalkaloids, whose carbon skeletons are derived from a mevalonic acid backbone and nitrogen sources are β-aminoethanol, ethylamine, and methylamine instead of amino acids. Terpenoid alkaloids include mono-, di-, and sesquiterpene alkaloids; amongst which diterpene alkaloids are the most important, as they are prominently used for pharmaceutical purposes. A less explored class of alkaloids includes chromone and flavoalkaloids, in which the nitrogen system is linked to “A” ring of chromone. In case of chromone alkaloids, chromone nucleus exists as noreugenin (5,7-dihydroxy-2-methylchromone), whereas flavoalkaloids bear an aryl substituent in the *C-2* position [27]. Flavonoids including flavons, flavonols, flavanones, and flavan-3-ols are present in the structure of flavoalkaloids, making this an important class due to their distinctive amphoteric nature (basic and phenolic) as well as their biological properties.

### 2.2. Chemical Structure

In this category, alkaloids are grouped under heterocyclic and non-heterocyclic compounds based on the position of the nitrogen atom in their chemical structure. In the heterocyclic group, nitrogen is present in the main heterocyclic ring, as is the case in alkaloids derived from L-tyrosine, L-phenylalanine, L-ornithine, L-tryptophan, L-lysine, and L-histidine, which are formed by the decarboxylation process of respective amino acid precursors. If a nitrogen atom occupies a position other than in the cyclic ring and is present in the aliphatic chain, non-heterocyclic alkaloids are formed. This non-heterocyclic group includes phenylethylamine- and tropolone-derived alkaloids, such as ephedrine, capsaicin, colchicine, and paclitaxel as main alkaloids (Table 1).

### 2.3. Taxonomy

Alkaloids produced by plant species of same genera are grouped under one category and this leads to broadened knowledge regarding the distribution of alkaloids in different plant species (Table 1). For instance, five main alkaloids including morphine, codeine, noscapine, thebaine, and papaverine produced in raw *Papaver somniferum* L. are grouped under opium alkaloids [6]. Solanum alkaloids include steroidal alkaloids and their corresponding glycosides present in the *Solanum* plant species, including potato, tomato, eggplant, and various nightshades [5]. Steroidal alkaloids and their glycosides include solanine, solasodine, solanidine, chaconine, tomatidine, tomatine, etc. Steroidal alkaloids are also found in the *Veratrum* genus, grouped as Veratrum alkaloids, including toxic veratridine, cyclopamine, and jervine [43].

Daphniphyllum alkaloids are structurally unique and diverse organic compounds produced in the plants of genus *Daphniphyllum* [26]. These alkaloids are derived from six molecules of mevalonic acid through a squalene-like intermediate and are divided into six nitrogen heterocyclic skeleton types, namely daphniphylline, secodaphniphylline, daphnilactone-A, daphnilactone-B, yuzurinine, and daphnigracine. An important group of alkaloids include *Vinca* alkaloids derived from *Catharanthus roseus*, which include leurosine, vinblastine, and vincristine. Vinblastine and vincristine are chemotherapeutic agents for cancer treatment. Their dimeric chemical structures are composed of two basic multi-ringed units; an indole nucleus (catharanthine) and a dihydroindole nucleus (vindoline) that are joined with other complex systems (Figure 1) [44]. Protoberberine alkaloids are another distinct class distributed among different genera of many plant families, including Annonaceae, Apocynaceae, Aristolochiaceae, Fabaceae, Lauracreae, Magnoliaceae, Menispermaceae, Ranuculaceae, Rutaceae, Berberidaceae, and Papaveraceae. These plant families comprise the highest number of plant species producing protoberberine alkaloids such as berberine, jatrorrhizine, and palmatine [45]. These are tetracyclic alkaloids derived from benzylisoquinolines by the process of phenolic oxidation and coupling with the isoquinoline N-methyl group, resulting in the formation of “berberine bridge” carbon. Plant species from the genus *Ephedra* produce phenylalanine-derived alkaloids ephedrine, pseudoephedrine, phenylpropanolamine, and cathine [46]. Of these ephedra alkaloids, ephedrine is the most potent thermogenic agent.

## 3. Chemical Reactions Involved in the Structural Modification of Plant Alkaloids

**Figure 2:**
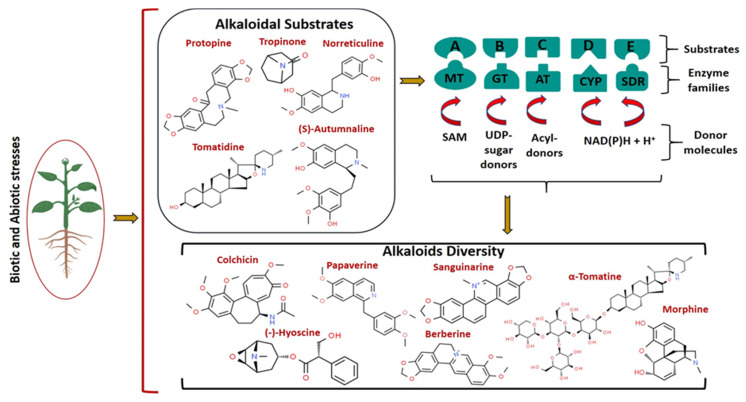
Alkaloid diversity in the plant kingdom. Alkaloids are produced in various parts of plants (such as the leaves, roots, seeds, etc.). Alkaloids are transported to required tissues mainly in response to various stress signals perceived from the environment. In the biosynthesis of alkaloids, enzymes of different families (MT: methyl transferase; GT: uridine-diphosphate-glycosyl transferase; AT: acyl transferase; CYP: cytochrome P450-monooxygenase and -reductase; SDR: short-chain dehydrogenase/reductase) act on alkaloidal substrates to generate diverse alkaloids with certain chemical modifications in numerous plant species. These enzymes catalyze the modification of alkaloidal substrates represented as A, B, C, D, and E in the presence of donors. SAM-*S*-adenosyl methionine acts as methyl donor for MTs; UDP-Glucose (UDP-Glc), UDP-rhamnose (UDP-Rha), UDP-xylose (UDP-Xyl), UDP-galactose (UDP-Gal), and UDP-glucuronic acid (UDP-GlcUA) are sugar donors for UDP-GTs; acyl-CoA thioesters and 1-*O*-β-glucose esters are acyl donors for ATs; NAD(P)H acts as an electron donor for CYPs and SDRs. Plant image is retrieved from BioRender (BioRender.com) (accessed on 20 March 2021). The chemical structures of alkaloids are drawn from “ChemSpider: the Free Chemical Database”.

**Figure 3:**
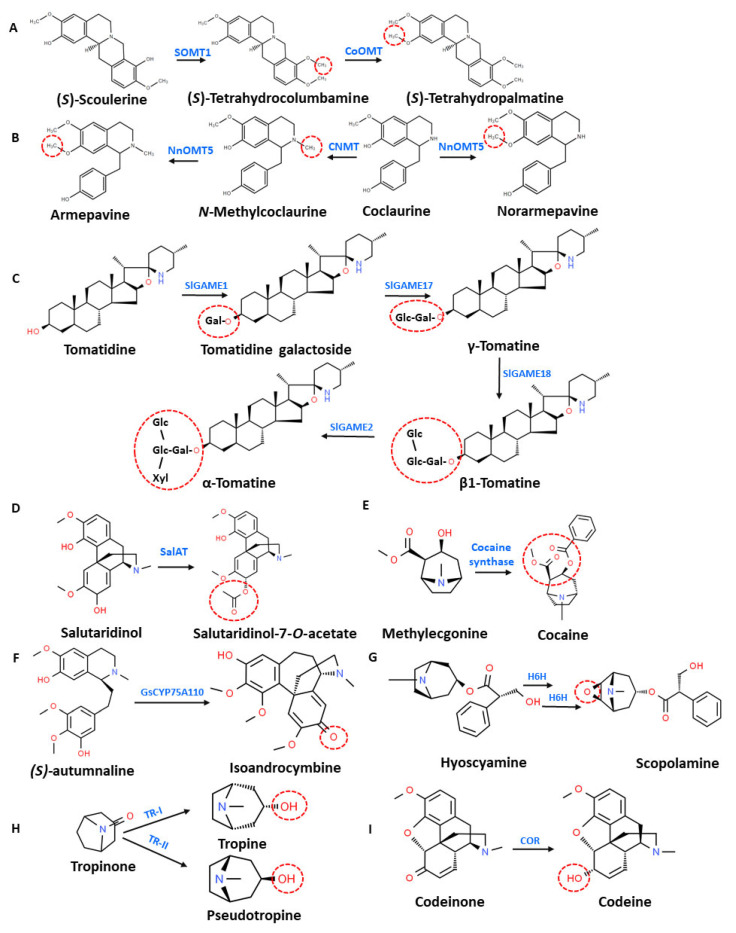
Examples of chemical reactions taking place in alkaloid biosynthesis in plants. Enzymes catalyzing various reactions are indicated in blue. Red dotted circles indicate the addition of functional entities at the respective positions. (**A**) Methylation reactions in the biosynthesis of alkaloids in genus Corydalis, *SoOMT1*: scoulerine 9-*O*-methyltransferase, *CoOMT*: columbamine *O*-methyltransferase; (**B**) Methylation reactions in the biosynthesis of alkaloids in Sacred lotus, *NnOMT5*: *O*-methyltransferase 5, *CNMT*: coclaurine *N*-methyltransferase; (**C**) Glycosylation reactions in the biosynthesis of steroidal glycoalkaloids in *Solanum lycopersicum*, *SlGAME*: glycoalkaloid metabolism; (**D**) Acetylation reaction in the biosynthesis of morphinan alkaloids, *SalAT*: salutaridinol 7-*O*-acetyltransferase; (**E**) Acetylation reaction in the biosynthesis of cocaine; (**F**) Oxidative para-para phenol coupling reaction in the biosynthesis of colchicine; (**G**) Hydroxylation reaction followed by epoxidation reaction in the biosynthesis of tropane alkaloids, *H6H*: hyoscyamine 6β-hydroxylase; (**H**) Reduction reactions catalyzed by two stereospecific reductases in the biosynthesis of tropane alkaloids, *TR-I/II*: tropinone reductase I/II; (**I**) Reduction reaction in the biosynthesis of morphinan alkaloids, *COR*: codeinone reductase. The chemical structures of alkaloids are drawn from “ChemSpider: the Free Chemical Database”.

Across the plant kingdom, modifying enzymes of different families act on many alkaloids to produce a diverse array of biologically important alkaloid derivatives having altered physical, chemical, and biological properties (Figure 2). Chemical modification reactions catalyzed by these enzymes, including mainly methylation, glycosylation, oxidation, reduction, hydroxylation, and acylation, are briefly described in this section. Few examples of these reactions in alkaloid biosynthesis in plants are depicted in Figure 3.

### 3.1. Methylation

Methylation plays a prominent role in developing the chemodiversity of alkaloids by functionalizing the parent compound with methyl groups. The methyl transferase (MT) gene family enzymes catalyze this type of reaction [47]. *S*-adenosyl L-methionine acts as the classical donor of methyl group for over 95% of MTs; which after utilization undergo SN2 nucleophilic reaction and are converted into *S*-adenosyl L-homocystine (SAH) with the addition of a methyl group to the alkylated substrate. The C-terminal domain of the enzyme carries a Rossmann fold for substrate binding and catalysis, whereas its N-terminal region plays a prominent role in dimerization. In plants, *O*-methyltransferase (OMT) and *N*-methyltransferase (NMT) are well-known classes of MTs catalyzing the methylation of alkaloids. OMT represents the largest class of enzymes catalyzing the methyl transfer reaction at the hydroxyl position of the alkaloidal substrate. The differential selectivity of substrates with respect to stereochemistry is an important feature displayed by plant OMTs, especially those involved in benzylisoquinoline alkaloids (BIAs) metabolism [48] (Figure 3A). For example, papaverine is an important BIA with antispasmodic activity. Several chemical modifications of intermediate alkaloids are reported during the biosynthesis of papaverine. Subsequent *O-*methylation steps of methylnorlaudanosoline by *4’OMT* and *N7OMT* produce *(S)*-norreticuline and *(S)*-norlaudanine, respectively. The methylation mediated by *3’-OMT* from the opium poppy (*Ps3’OMT*) has been implicated in the formation of two alkaloids, i.e., (*S*)-tetrahydropapaverine (a benzyltetrahydroisoquinoline alkaloid) and *(S)*-nororientaline (a tetrahydroisoquinoline alkaloid) [49].

In Sacred lotus (*Nelumbo nucifera* Gaertn), methylation reactions generate a variety of alkaloids belonging to aporphin and bisbenzylisoquinoline structural categories [50]. The methylation of norcoclaurine by NnOMT1 generates coclaurine, which is a nicotinic acetylcholine receptor antagonist having a tetrahydroisoquinoline structure similar to norcoclaurine [51]. Unlike OMT, NMT acts on nitrogen atom of substrate adding methyl group. The conversion of coclaurine to *N*-methylcoclaurine is catalyzed by coclaurine *N*-methyltransferase (NnCNMT1). NnOMT5 catalyzes 7-*O*-methylations of coclaurine and *N*-methylcoclaurine, generating norarmepavine and armepavine, respectively (Figure 3B) [51]. In *Laburnum anagyroides*, *S*-adenosyl L-methionine-dependent cytisine *N*-methyltransferase catalyzes the transfer of a methyl group from *S-*adenosyl L-methionine to cytisine, a quinolizidine alkaloid; the same enzyme further methylates cytisine to the less polar *N*-methylcytisine [52].

### 3.2. Glycosylation

Numerous glycosyl transferases catalyzing glycosylation reactions are reported in plants that transfer a glycosyl moiety from nucleotide sugar donors to different acceptor molecules such as hormones and specialized metabolites [53]. These enzymes show less similarity in their primary sequences, although a 44-amino acid sequence, named as plant secondary product glycosyltransferase (PSPG)-box, is highly conserved. This box present in C-terminal region is believed to be involved in binding to the UDP moiety. These enzymes catalyze a SN2 nucleophilic displacement reaction between nucleotide sugar donors such as UDP-Glucose (UDP-Glc), UDP-rhamnose (UDP-Rha), UDP-xylose (UDP-Xyl), UDP-galactose (UDP-Gal), and UDP-glucuronic acid (UDP-GlcUA) and nucleophile acceptors. The resulting glycosylated compounds alter biological, physical, and chemical properties from the parent compounds along with distinct subcellular localization (Figure 3C). The glycosylation process has been proven to be helpful in improving the stability, solubility, and toxicity modulation of plant specialized metabolites [54,55].

In cultivated potato plants, solanidine undergoes glycosylation reactions at the 3-OH position by a set of glycosyltransferases, including solanidine galactosyltransferase (SGT1), solanidine glycosyltransferase (SGT2), and rhamnosyltransferase (SGT3), forming α-solanine and α-chaconine steroidal glycoalkaloids (SGAs) [56,57]. However, in wild potato plants, these SGAs are converted into leptines by a glykoalkaloid metabolism encoding gene—*GAME32* [5]. The toxicity of the steroidal alkaloid tomatidine towards plant cells has been reported in tomato plants; therefore, to prevent self-toxicity, it is glycosylated with various sugar moieties in the presence of uridine diphosphate-glycosyl transferases encoded by *SlGAME1, -2, -17,* and *-18* to generate α-tomatine (Figure 3C) [58,59]. It is the major SGA produced in the green leaves and unripe fruits of tomato plants and provide protection to plants against a plethora of microorganisms.

The in vitro enzymatic activity of three new GTs (UGT84A33, UGT71AE1, and UGT90A14) from *Carthamus tinctorius* has been tested for the synthesis of BIA glycosides from BIAs like berberines, berberrubine, jatrorrhizine, columbamine, palmaturbin, groenlandicine, protoberberines, etc. [60]. The glycosylating potential of these GTs can be helpful in generating novel alkaloids glycosides with improved bioactivity and reduced toxicity.

### 3.3. Acylation

The process of acylation in several classes of specialized metabolites contributes to the generation of chemodiversity in compounds [61]. The acylation reaction involves the transfer of an acyl group from an activated donor to an acceptor molecule, catalyzed by acyltransferases (ATs). Two types of enzymes belonging to BAHD-acyltransferases (BAHD-ATs) and Serine CarboxyPeptidase-like (SCPL)-acyltransferases (SCPL-ATs) are known. These enzymes require “energy-rich” donor molecules; acyl-CoA thioesters serve as donors for BAHD-ATs, whereas SCPL-ATs utilize 1-*O*-β-glucose esters. The interplay between BAHD- and SCPL-ATs in different subcellular compartments is a necessary criteria for the secondary modification steps of different compounds. A cytosolic role has been reported for various members of BAHD-AT family [62,63]; however, in plants, the acyl-CoA thioester is synthesized in vacuoles that are transported to cytosol to serve as donors for BAHD-ATs. On the other hand, SCPL-ATs carry out the downstream processing of metabolites in vacuoles [64], however, the formation of 1-*O*-β-glucose esters is catalyzed by UGTs in cytosol. This glycosylation step generates an active substrate for SCPL-ATs by adding a glucose tag to enable recognition by the transporters required for importation from cytosol to vacuole.

In the biosynthesis of morphinan alkaloids, salutaridinol 7-*O*-acetyltransferase plays a crucial role in the acylation of phenanthrene alkaloid salutaridinol, utilizing acetyl-CoA as the donor to form salutaridinol-7-*O*-acetate (Figure 3D), an immediate precursor for thebaine [65]. In monoterpene indole alkaloids biosynthesis, the acetyl-CoA- or CoA-dependent reversible formation of vinorine (or 11-methoxy-vinorine) and 16-epi-vellosimine is catalyzed by vinorine synthase. These indole alkaloids act as a direct precursor for the ajmaline biosynthetic route. The tissue-specific expression patterns of BAHD-ATs have been reported, as in the case of the tabersonine derivative 19-*O*-acetyltransferase (TAT), which is able to acetylate minovincinine and horhammericine, the 19-hydroxytabersonine derivatives from roots [66]. In coca plants, cocaine synthase is an important enzyme belonging to BAHD family that catalyzes the condensation of a pharmacologically inactive alkaloid, methylecgonine with benzoyl-CoA derived from L-phenylalanine, to produce cocaine, which is a powerful stimulant [61] (Figure 3E). Interestingly, littorine is a tropane alkaloid, which is structurally similar to cocaine, and both involve an esterification reaction in the last step of their respective biosynthesis pathways. However, this reaction is catalyzed by different enzymes in both cases, e.g., cocaine synthase catalyzes the formation of cocaine in coca plants [61], whereas littorine synthase belonging to SCPL family condenses phenyllactylglucose and tropine via esterification to form littorine in Solanaceous plants [67].

### 3.4. Oxidation

Oxidation reactions taking place along the biosynthetic route of specialized metabolites occur in a stereo- and regio-specific manner. The formation of a diverse range of alkaloids and their parent compounds involves multiple oxidation reactions utilizing aromatic amino acid precursors. These reactions are catalyzed by cytochrome P-450 (CYP) enzymes, 2-oxoglutarate-dependent dioxygenases, and flavoproteins. CYPs containing haem as cofactor are a superfamily of enzymes exhibiting a broad diversity in their chemical structure and biological functions, occurring in different families and subfamilies across the plant kingdom [68]. CYP450 monooxygenases perform hydroxylation reactions, which are the most common type of oxidation reactions in alkaloid formation. In Sacred lotus, two CYP450 monooxygenases belonging to CYP80G and CYP719A families are proposed to catalyze the conversion of *N*-methylcoclaurine to aporphins such as lirinidine and roemerine [69]. The C–O coupling reaction required for the conversion of *N*-methylcoclaurine into Nelumboferine (a bisbenzylisoquinoline alkaloid) is possibly catalyzed by an enzyme encoded by the *CYP80A* family [70].

In SGA biosynthesis, in Solanaceous plants such as tomato and potato, hydroxylation and oxidation reactions are carried out by *GAME* genes that encode for CYP450 monooxygenases [59]. These harmful SGAs are converted into non-toxic specialized metabolites by a series of chemical modifications involving hydroxylation, acetylation, and glycosylation. In tomato, the 2-oxoglutarate-dependent dioxygenase (2-ODD) enzyme encoded by *GAME31* catalyzes the hydroxylation of bitter α-tomatine to hydroxytomatine during ripening, which is an important step towards the formation of non-bitter esculeosides [5]. In potato, this enzyme is encoded by *GAME32*, which hydroxylates bitter SGAs to produce leptinines, which further produce leptines responsible for providing protection to plants against the Colorado potato beetle [5].

In the formation of colchicine in *Colchicum* and *Gloriosa* plants, *(S)-*autumnaline undergoes an oxidative para-para phenol coupling reaction catalyzed by GsCYP75A110 to form another isoquinoline alkaloid, namely isoandrocymbine (Figure 3F). The methylation of this compound by GsOMT4 yields *O*-methylandrocymbine. Furthermore, the expansion of the dienone ring of the previous compound and the formation of a tropolone ring takes place. These steps are catalyzed by GsCYP71FB1, which forms a tropolone-containing compound, N-formyldemecolcine [35].

For tropane alkaloids biosynthesis, the condensation of tropine with activated *(R)*-phenyllactate delivers the third ring intermediate to form littorine by littorine synthase. Further rearrangement by littorine mutase (a cytochrome P450) produces hyoscyamine aldehyde, which reduces to hyoscyamine [71]. The enzyme hyoscyamine 6β-hydroxylase is a 2-oxoglutarate dependent dioxygenase showing bifunctional properties. Firstly, it carries out the hydroxylation of hyoscyamine to 6β-hydroxy hyoscyamine, and secondly, the epoxidation of 6β-hydroxy hyoscyamine to scopolamine [72] (Figure 3G). These alkaloids possess differences in biological effects despite having a similar tropane ring structure [32].

The final step of papaverine biosynthesis involves the activity of *TPOX* (tetrahydropapaverine oxidase), which dehydrogenates the *O*-methylated and *N*-desmethyl alkaloid tetrahydropapaverine to yield papaverine [73]. Expanding the papaverine biosynthetic pathway, a novel 2-oxoglutarate/Fe_2_+-dependent dioxygenases (2ODD) catalyzing the efficient substrate- and regio-specific 7-*O-*demethylation of papaverine yielding pacodine (analogue of papaverine) has been reported [74].

### 3.5. Reduction

The cytochrome P450 reductase (CPR), short-chain dehydrogenase/reductase (SDR), and aldo-keto reductase (AKR) superfamilies are enzymes that carry out reduction reactions to several alkaloids. In the formation of tropane alkaloids in Solanaceae plants, the reduction of the keto group in the tropane ring is catalyzed by stereospecific tropinone reductases (TRs), which are NAD (P)(H)-dependent monomeric oxidoreductases belonging to the SDR enzyme family [75]. Pathways to two distinct tropane alkaloid categories, scopolamine and calystegines, are decided upon by two reductases [72]. Tropinone reductase I coverts tropinone to tropine (3α-tropanol), which is used to produce scopolamine. Tropinone reductase II reduces tropinone to pseudotropine (3β-tropanol) (Figure 3H), which further proceeds towards calystegine biosynthesis.

A short-chain alcohol dehydrogenase/reductase co-expressing with norbelladine 4’-*O*-methyltransferase from *Narcissus* and *Galanthus* spp. catalyzes a carbon-carbon double bond reduction in noroxomaritidine to form oxomaritinamine, which is required for the biosynthesis of amaryllidaceae alkaloids [76]. In several cases, CYP450 enzymes require the shared activity of CPR for two-electron transfer activity. It was studied in *C. roseus* that a class II CPR provides electrons for highly expressing P450s, which are involved in tissue-specific and induced specialized metabolism [77]. The cloning and purification of first plant CPR was reported in *C. roseus* [78,79]. Perakin reductase is the example of first aldo-keto reductase superfamily enzyme, isolated from *Rauvolfia* sp., and was found to be involved in monoterpene indole alkaloid biosynthesis [80].

## 4. Multiple Chemical Conversions in Plant Alkaloids

A series of biochemical modification reactions in plants bring with them new sequels of alkaloids with diverse arrays of chemical structures and biological activities. In this section, we have highlighted some plant alkaloids involving multiple chemical modifications that occur in the final steps of alkaloidal conversions.

### 4.1. Noscapine Alkaloids

The diverse class of the isoquinoline alkaloid contains noscapine as an important non-narcotic drug belonging to the phthalideisoquinoline subclass, which has been isolated from the Papaveraceae family plant species. Noscapine undergoes 3′-hydroxylation and a series of *O*- and *N*-methylations to form (*S*)-reticuline, which is one of the alkaloids found in the opium poppy. Methylene-bridge formation takes place in (*S*)-reticuline via the berberine bridge enzyme (BBE) to form the protoberberine alkaloid (*S*)-scoulerine, which undergoes 9-*O*-methylation catalyzed by an enzyme encoded by *S9OMT1* to form *(S)-*tetrahydrocolumbanine. *Canadine synthase* gene encoding CYP719A19 catalyzes the formation of the methylenedioxy bridge and generates *(S)*-canadine, which can block K (ATP) channels in dopamine neurons [81]. Further, the oxidation of a cyclic narcotine hemiacetal (a benzylisoquinoline alkaloid and a cyclic acetal) to the lactone ring in noscapine is performed by noscapine synthase (NOS), also known as short-chain dehydrogenase/reductase (SDR1).

### 4.2. Morphinan Alkaloids

Another important class of BIAs in opium poppy includes morphinan alkaloids, which are strong narcotic analgesics. These comprise of natural opiates like morphine and codeine and their semisynthetic derivatives, such as dihydromorphine and hydromorphone [6]. These are used for treating severe pain associated with cancer, rheumatism, and dental problems. The morphine pathway diverges from other BIA pathways as it utilizes *(R)-*reticuline instead of *(S)-*reticuline [82]. The conversion of *(S)-*reticuline to *(R)*-reticuline is catalyzed by STORR ([*S*]-to [*R*]-reticuline), which is a P450 enzyme displaying separate domains for two enzymatic activities [74]. In the first part of reaction, the cytochrome P450 module of the enzyme converts *(S)*-reticuline to 1,2-dehydroreticuline, and in the latter part, oxidoreductase module converts 1,2-dehydroreticuline to *(R)*-reticuline. The salutaridine, which is the basic skeleton of opiates, is formed by the coupling reaction between the 20th position and 10th position of the carbon atoms of (R)-reticuline catalyzed by salutaridine synthase. Salutaridine reductase performs the reduction of salutaridine to produce thebaine, followed by acetylation with salutaridine 7-*O*-acetyltransferase and a spontaneously occurring deacetylation reaction [65]. Thebaine undergoes demethylation by thebaine 6-*O*-demethylase and reduction by codeinone reductase to produce codeine (Figure 3I). Morphine is synthesized by the demethylation of codeine by codeine *O*-demethylase.

### 4.3. Sanguinarine, Protopines, and Berberine Type Alkaloids

Sanguinarine is a benzophenanthridine alkaloid, belonging to the BIA class of alkaloids extracted from many plant species, such as *Sanguinaria canadensis*, *Chelidonium majus,* and *Macleaya cordata* [83]. The BIA alkaloid (*S*)-Scoulerine undergoes several modification reactions to generate a series of alkaloids possessing diverse biological properties. (*S*)-Scoulerine is synthesized from *(S)*-reticuline by the berberine bridge enzyme [6]. Cheilanthifoline synthase, which is a member of the CYP719A subfamily, catalyzes the addition of a methylenedioxy bridge to form *(S)-*cheilanthifoline, which is used in Bhutanese traditional medicine for the treatment of fever [84]. Further, the oxidation of *(S)-*cheilanthifoline is performed by another CYP719A enzyme, stylopine synthase, to form *(S)-*stylopine. Opium poppy TNMT catalyzes the *N*-methylation of *(S)-*stylopine to form *(S)-cis-N-*methylstylopine, which is further acted upon by *N*-methylstylopine 14-hydroxylase, a member of the CYP82 N subfamily, to produce protopine. Protopine acts as an analgesic and also inhibits histamine H1 receptors and platelet aggregation [85]. Protopine undergoes 6-hydroxylation and gets converted into dihydrosanguinarine by *P6H* protopine 6-hydroxylase. *DBOX (*dihydrosanguinarine oxidase) and *SanR* (sanguinarine reductase) catalyze forward and backward reactions to produce sanguinarine, which is a toxic alkaloid [73]. STOX, (*S*)-tetrahydroxy protoberberine oxidase, produces berberine, which is a protoberberine type of isoquinoline alkaloid. Berberine shows antimicrobial activity and antidiabetic effects in experimental animal and clinical diabetic patients.

### 4.4. Monoterpene Indole Alkaloids

The biological activities attributed to this group of alkaloids make them promising candidates for utilization in the pharmaceutical industry [44]. Stemmadenine is an alkaloid which after second carbon-carbon cleavage forms dehydrosecodine (an acrylic ester). This intermediate possibly goes through a Diels Alder reaction towards an iboga-type alkaloid, catharanthine, and an aspidosperma-type alkaloid, tabersonine. Tabersonine is converted into vindoline by via seven-step pathway involving hydroxylation, *O*-methylation, C-3-oxidation, C-2/C-3-reduction step, *N*-methylation, C-4-hydroxylation, and C-4-O-acetylation [86]. Vindoline is the precursor for important the anticancerous metabolites, vincristine and vinblastine. The synthesis of these metabolites takes place in the leaf tissues of the plant.

In the roots of *C. roseus* and *Tabernaemontana divaricata,* lochnericine is a major MIA derived from the stereoselective C6, C7-epoxidation of tabersonine by tabersonine 6,7-epoxidase 1. In another biosynthetic route, *O*-acetylstemmadenine is acted upon by vincadiformine synthase 1 or 2 (VS1/2) to form vincadiformine, which undergoes hydroxylation to form minovincinine by vincadiformine 19-hydroxylase. This is followed by acetylation by minovincinine 19-hydroxy-*O*-acetyltransferase (MAT) to form echitovenine [38,87]. In the contrasting biosynthetic route, the hydroxylated form of vincadiformine is methylated by 16-*O*-methyltransferase to form ervinceine [88]. Vincadiformine and vincamine are reported at higher levels in the leaves of *Vinca minor* [89]. Vincamine is synthesized in leaves of *V. minor* when 16-methoxy tabersonine undergoes epoxidation in the presence of tabersonine 3-oxidase (T3O), which after rearrangement forms an eburnamine-vincamine like skeleton (fundamental parent alkaloid) that further transforms into vincamine [90].

## 5. Role of Alkaloids in Plant Defense against Biotic and Abiotic Stresses

**Figure 4:**
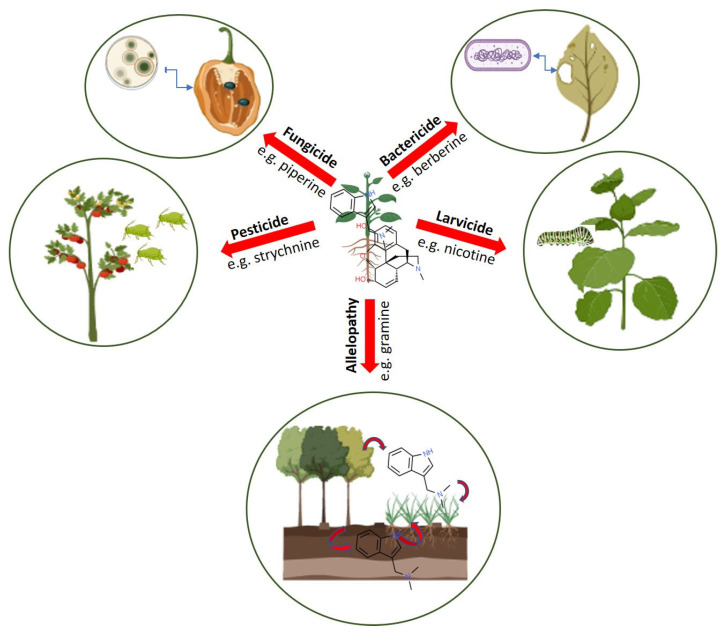
The role of alkaloids in plant defense. Alkaloids produced in different plant tissues, such as leaves, roots, bark, and seeds, are transported to local tissues for fighting against various predators such as pests, fungi, bacteria, and insect larvae, providing plants with protection against these predators. Alkaloids also stop the growth of other plants in the vicinity (allelopathy). Individual images of plant and predators are retrieved from BioRender (BioRender.com) (accessed on 20 March 2021).

Alkaloids act as reservoirs for nitrogen storage and plants have evolved their metabolic diversity to cope with environmental stress conditions. For example, plants use specialized metabolites as an integral part of their defense system, including biotic and abiotic stress responses (Figure 4). Furthermore, these specialized metabolites are found in varying levels in different tissues of plants (e.g., leaf, stem, root, flower, seed, fruit, and storage organs), and offer protection against a diverse variety of pests, predators, and herbivores. Synthesized alkaloids are stored in specific cellular compartments and upon sensing different stress signals from environment they are released from the stored organelle/specific glands and exported to the target tissues.

Many alkaloids show potent antimicrobial activities against various pathogenic microorganisms. Specific alkaloids, such as α-tomatine from tomato, piperine from black pepper, and protoberberines and berberines from a wide range of plant species including *Berberis aristate*, exhibit both antimicrobial and antifungal properties [91,92]. However, selective alkaloids possess only antibacterial activities, for example, squalamine acts against *Klebsiella pneumoniae*, lysergol acts against *Escherichia coli*, and tomatidine acts against *Staphylococcus aureus*, *Bacillus cereus*, *B. subtilis*, and *Listeria monocytogenes* [93]. Some alkaloids specifically exhibit antifungal properties, such as the tomatidine glycoalkaloid from tomato acts which against yeast—*Saccharomyces cerevisiae* [94], quinoline from *Waltheria indica* L. which acts against *Candida albicans* [95], and Jatrorrhizine (a protoberberine alkaloid from *Mahonia aquifolium*), β-carboline, and cocsoline which act against several fungal species [96,97]. Other alkaloids (such as perivine, leurocristine, berberine, and acrimarine F) show antiviral activities against poliovirus, vaccinia, influenza, HIV, and Epstein-Barr virus [98] (Figure 4).

**Figure 5:**
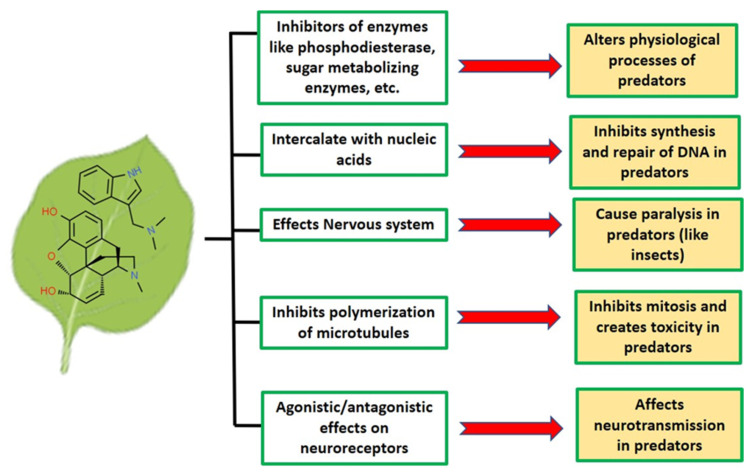
The mechanism of action of alkaloids. Alkaloids act as a chemical barrier that protects plants from predators, such as herbivorous insects and vertebrates; pathogenic bacteria and fungi; and parasitic plants. Various mechanisms employed by plants using alkaloids create harmful effects in predators and are depicted in the figure.

Several alkaloids such as nicotine, α-chaconine, and α-solanine are known to possess toxicity against various chewing insects (e.g., *Spodoptera exigua, Manduca sexta*, and *Tecia solanivora*) as well as sucking insects such as whiteflies, aphids, and planthoppers [99] (Figure 4). The aphicidal activity of alkaloids isolated from Amaryllidaceae plants has been reported against *Aphis citricola*. Colchicine produced by *Colchicum autumnale* inhibits the polymerization of tubulin and the depolymerization of microtubule during mitosis, and causes toxicity to predators such as the honeybee (*Apis mellifera*) and the honeycomb moth (*Galleria mellonella*) [100] (Figure 5).

Nitrogen-fixing plants, as well as plants grown on high-nitrogen contents, accumulate high levels of alkaloids in their leaves making htem more resistant to herbivory. Alkaloids that are derived from aromatic amino acids (e.g., isoquinoline, quinoline, and indole alkaloids) are known to exhibit antiherbivoral activities. Many alkaloids act on different enzymes of predators to disturb their physiological processes (Figure 5). Swainsonine from locoweed (*Astragalus* and *Oxytropis* species) is known to inhibit the activity of α-mannosidase, and thus, affects the synthesis of N-glycans in cellular membranes and the ingestion of respective plants causes intoxication amongst livestock [101]. Alkaloids like morphine, codeine, and caffeine produce stimulatory effects on the central nervous system of predators, which can cause paralysis attack in predators (Figure 5). Some alkaloids are highly poisonous to mammals and other animals. For example, plants containing strychnine, brucine, and atropines upon ingestion by various predators produce serious effects on neurotransmission and the central nervous system, leading to the death of individuals (Figure 5). Specific plant alkaloids, such as quinine, emetine, β-carboline, furanocoumarin, and furanoquinoline, have nucleic acid intercalating properties, interfereing with DNA replication and repair mechanisms, which may lead to mutations and genotoxicity [102]. Overall, alkaloids display distinct mechanisms to protect plants from predator attack.

Furthermore, certain alkaloids leached from leaves, roots, and other plant tissues exhibit allelopathic effects by affecting the growth potential of the roots and shoots of other competitor plants (Figure 4). For example, capsaicin, the pungent alkaloid produced mostly in the seeds of *Capsicum* species, has been shown to affect the germination, seedling growth, and chlorophyll accumulation in mung bean (*Vigna radiata*) plants [103]. Several other alkaloids such as berberine, gramine, and sanguinarine exhibit allelopathic effects against *Lactuca sativa* and *Lepidium sativum* seedlings [7] (Figure 4).

Various abiotic stress conditions such as drought, salinity, high temperatures, etc., are also known to influence the accumulation of alkaloids in many plant species. For example, drought stress alters the levels of chinolizidin alkaloids in *Lupinus angustifolius* [104] and morphine alkaloids in *Papaver somniferum* [105]. Moreover, emerging studies suggest that heat and drought conditions alone or in combination can alter the accumulation of alkaloids in plant species such as *Mentha piperita* and *C. roseus* [106]. Tropane alkaloid levels in the young leaves of *Datura innoxia* could be induced by salt stress (153.8 moL/m^3^ NaCl) [107]. Using a B5 suspension culture of *C. roseus*, it has been demonstrated that the total alkaloid yield could be enhanced during salinity stress (100 mM NaCl) [108]. An increased alkaloid content in *C. roseus* seedlings exposed to salt or salinity stress has also been reported previously [109]. The effects of different nitrogen sources on the levels of indole alkaloid content in *C. roseus* seedlings has also been studied [110], wherein potassium nitrate (20 mM KNO_3_) treatment led to an increased accumulation of alkaloid content in leaves compared to ammonium chloride (2 mM NH_4_Cl). Moreover, it was earlier reported that UV-B significantly increased the content of terpenoid indole alkaloids (lochnericine and ajmalicine) in the hairy roots of *C. roseus* [111]. Apart from abiotic factors, phytohormones are also known to influence the levels of alkaloids in plants [112,113].

## 6. Biological Activities of Alkaloids and Their Potential Applications with Special Reference to Therapeutics and Pharmacology

Since ancient times, alkaloids have shown great effects on animal and human lives and are an inclusive part of the food and beverages consumed in daily life. Besides, these compounds are used in medicinal and stimulant drugs with core biological activities (Table 2). Alkaloids with anticancerous activities, such as vincristine, vinblastine, and taxol, are being effectively used as chemotherapeutic drugs. Vincristine and vinblastine isolated from *Vinca rosea* act by binding to tubulin; however, they work on different tumor types. Vincristine is used to treat acute leukemia and other lymphomas, while vinblastine is mainly used for the treatment of Hodgkin lymphoma and advanced breast or testicular cancer [114]. Other Vinca alkaloids, vinorellaine and vinflunine, are used to treat lung cancer and urothelial cancer, respectively [115]. Berberine, an isoquinoline alkaloid, has been shown to possess anticancer potential through the inhibition cell proliferation by interacting with respective microRNAs and suppressing telomerase activity [116,117] (Table 2). Evodiamine, the bioactive compound isolated from *Evodia rutaecarpa,* is a quinolone alkaloid which has been shown to exhibit anticancer activities both in vitro and in vivo by inhibiting angiogenesis, invasion, and metastasis in a variety of cancer cell lines [118]. Piperine is an alkaloid from *Piper nigrum* and *P. longum* which is shown to exhibit chemopreventive effects. This dietary phytochemical can act against several kinds of carcinogen, such as 7,12-dimethyl benz(a)anthracene and benzo(a)pyrene [119]. Colchicine extracted from Colchicum and Gloriosa plant species has been approved by the Food and Drug Administration (FDA, USA) for the treatment of acute cases of gout Mediterranean fever, pericarditis, and Behcet’s disease [120,121]. The bioactivity of colchicine is thought to result from its interaction with tubulin dimers and the subsequent inhibition of microtubule growth [122]. However, colchicine alone or in combination with taxane and Vinca alkaloids is too potent to be used in chemotherapy [120] (Table 2).

The Vinca alkaloid category includes vincamine, which has vasodilatory activity and increases blood flow to the brain. It is sold in tablet form in Europe and has also been used as a nootropic supplement in diet to improve the brain function of healthy people [90]. Some alkaloids show psychotropic effects by stimulating the central nervous system. These include cocaine, ephedrine, and strychnine, and their long-term use can cause addiction and serious adverse effects upon frequent consumption. Cocaine is frequently used as a recreational drug due to its stimulant property, causing mental effects of feelings of joy [148]. Aside from harmful effects, a central nervous system stimulant such as ephedrine has a bronchial smooth muscle relaxant property, and therefore, it is used as a decongestant [149]. Strychnine, a terpene indole alkaloid, is a highly toxic compound to humans as well as other vertebrate animals, such as rats and birds. It is mainly used as a rodenticide but varies in specificity and can kill other animals too [124]. Quinazoline alkaloids such as vasicinone and vasicine show bronchodilatory activity and are respiratory stimulant used in various asthma medications [126].

Alkaloids also have the potential to reduce hypertension. Tetrandrine isolated from *Stephania tetrandra* has been reported to be useful for the treatment of hypertension in patients accompanied with poor sleep efficiency [129]. Reserpine and Ajmalicine are recommended alternative drugs for treating hypertension [128]. Alkaloids extracted from the opium poppy (*P. somniferum*) induce analgesic and narcotic effects by acting upon opioid receptors. The alkaloids from poppy include morphine, codeine, thebaine, noscapine, and their derivatives, which are used for treating moderate to severe pain. However, they may cause adverse side effects such as dizziness, sedation, nausea, vomiting, respiratory depression, dependency, and tolerance [150,151]. Noscapine has been used as safe cough suppressant and has evoked attention of pharmaceutical industries in cancer treatment (Table 2).

*(S)*-reticuline, the central pathway intermediate of most BIAs, is a potent central nervous system depressant and is also suggested to be responsible for atypical parkinsonism [152]. Hyoscyamine extracted from *Datura stramonium* is an antagonist of muscarinic acetylcholine receptors and can control neuropathic pain; the efficacy of its analgesic effect is improved when used in combination with opioids [153]. Scopolamine, also known as hyoscine, is actively produced in *Hyoscyamus niger* and has a strong hallucinogenic effect, making it a constituent of psychoactive drugs [32]. Veratrum alkaloids are toxic compounds that can cause rapid heart failure by activating sodium ion channels. Although veratrum alkaloids are toxic, they have been used for the treatment of myasthenia gravis and hypotension [139] (Table 2).

Another alkaloid in the protoberberine group is coptisine, which is used in Chinese herbal formulations. Coptisine exhibits a wide range of pharmacological properties such as antibacterial, hypoglycemic, anti-tumerogenic, and neuroprotectant effects. Palmitine is the major component of the alkaloidal extract of *Enantia chlorantha,* which has been studied for its use in the treatment of jaundice, hypertension, inflammation, dysentery, and liver-related diseases. Anti-inflammatory, antimicrobial, and antifungal activities have been reported for jatrorrhizine, which is also an important alkaloid in the above protoberberine-type alkaloidal group. Ephedrine and pseudoephedrine are used in decongestants and cold medicines. Ephedrine-related Chinese formulations are sold as dietary supplements for effective weight loss and to enhance athletic performance. Cytisine, a quinolizidine alkaloid from *Laburnum* and *Cytisus* plants in the Fabaceae family, acts as a partial agonist of nicotinic acetylcholine receptors (nAChRs) and has been used to help smoking cessation [147] (Table 2).

Steroidal alkaloids and their glycosides present in Solanaceous plants pose various biological activities ranging from toxic to useful properties. α-tomatine produced in the green tissue of tomato plants is an antinutritional compound and its consumption in the range of 500 to 5000 mg/kg of dry weight in green tomato recipes shows toxicity in humans [154]. On the other hand, tomatine can act as a powerful adjuvant, and is reported to elicit an antigen-specific cell-mediated immune response to a pre-erythrocytic stage malaria vaccine candidate antigen [155]. α-chaconine present in green potatoes is a natural toxicant which causes harmful physiological effects in other organisms but provides protection to plants against fungi and insects [5].

Aporphin and bisbenzylisoquinoline alkaloids from Sacred lotus possess nutritional and medicinal values. Norcoclaurine, also known as higenamine, obtained from lotus seeds is reported to show notable pharmacological properties such as anti-inflammatory, anti-thrombotic, and β-adrenergic receptor agonist effects [156]. Norarmepavine and armepavine have potential to be used in the cosmetic industry due to their melanogenesis inhibition activity [157]. Due to the immunomodulatory effect of armepavine, it has been used as potent herbal drug in the treatment of autoimmune disorders such as systemic lupus erythematosus and crescentic glomerulonephritis [158]. Roemerine has been ascribed anti-fungal and anti-malarial properties. Nelumboferine, along with other bisbenzylisoquinoline alkaloids, such as neferine and liensinine, exhibited sedative effects in a mouse model [70] (Table 2).

Hundreds of alkaloids have activities against bacteria (e.g., squalamine, lysergol, tomatidine, etc.); fungi (e.g., tomadine, quinoline, β-carboline, and cocsoline, etc.) and viruses (e.g., leurocristine, periformyline, perivine, and vincaleucoblastine) [98]. Thus, the antibiotic property of alkaloids has been efficiently utilized by human beings for pharmaceutical purposes. The commercialization of products containing alkaloids has created new opportunities in the market to treat various health ailments (Table 3). Many of them also act as stimulant drugs (e.g., nicotine, morphine, caffeine, codeine, etc.). The nutraceutical values of alkaloids have been employed for manufacturing dietary ingredients (e.g., caffeine, atropine, and cocaine), nutritional supplements in combination with other natural compounds (e.g., hyoscyamine, scopolamine, tigloiodine, and cocaine), as well as natural food preservatives [159] (Table 3).

## 7. Conclusions and Future Prospects

Plants have evolved with structural and chemo-protective shields to combat numerous environmental stress conditions. As a defensive mechanism, alkaloids participate in various guises, such as in attacking insects, herbivores, and pathogens; thus, protecting the plants against different biotic stress conditions. The availability of precursors, enzymes required for catalysis, and chemical modifications in alkaloidal transformations have given rise to structural diversity in alkaloids. Enzymes encoded by various gene superfamilies have evolved with the ability to modify a broad range of metabolites. The altered chemical and biological properties of alkaloids are the outcome of the arrangement of chemical moieties in a diversified fashion. With the aim to comprehend this broad and diverse class of specialized metabolites, we have briefly described the classification of alkaloids according to their biosynthetic, chemical, and taxonomical aspects. The major emphasis of this review is on the structural modifications in alkaloidal inter-conversions in different plants species and their applications in plant defense, as well as human therapeutics and pharmacology. The enzymes catalyzing the biochemical transformation of some of the commercially important alkaloids such as vasicinone, ephedrine, piperine, lupines, and other specialized metabolites are yet to be accomplished. In known cases, these enzymes are highly specific for the substrate as well as for the formation of products. Transcriptomic and genomic approaches coupled with the metabolomic data would be helpful in generating deep insights about the structural diversity and bioactivity of commercially important alkaloids. Interestingly, in several cases, the alkaloid biosynthetic pathway genes in plants are clustered on specific chromosomes [59,160]. Such features can be further explored through advanced genome editing tools for the higher production of desired alkaloid(s) in plants [161].

## Figures and Tables

**Table 1:** Classification of alkaloids. Alkaloids biosynthesized in plants are classified based on their biochemical precursors (biosynthetic pathway), chemical structures, and according to their occurrence in different genera of the plant kingdom.

Group	Characteristic	Representative Compounds	Plant Source	Reference
	Feature			
**Biosynthetic Pathway—Alkaloids Biosynthesized from Common Precursor**				
**Tetrahydroisoquinoline alkaloids**	Tyrosine acts as precursor	Berberine	*Argemone mexicana, Berberis aristata, B. aquifolium*, *B. heterophylla*, *B. beaniana*, *Coscinium fenestratum*, *C. chinensis*, *C. japonica*, *C. rhizome, Hydratis Canadensis*, *Chelidonium majus, Coptidis rhizome*	[9]
**Indole alkaloids**	Tryptophan acts as precursor	Ajmalicin	*Rauvolfia* spp., *Catharanthus roseus*, *Mitragyna speciosa*	[10,11,12]
		strychnine, brucine	*Strychnos nuxvomica*	
**Pyrrolizidine alkaloids**	Ornithine acts as precursor	Senecionine	*Jacobaea vulgaris, Brachyglottis repanda, Emilia* sp., *Erechtites hieraciifolius, Petasites* sp., *Syneilesis* sp., *Crotalaria* sp., *Senecio* sp., *Cynoglossum* sp., *Symphytum* sp., *Heliotropium* sp., *Caltha leptosepala*, *Castilleja* sp.	[13,14]
**Tropane alkaloids**		Scopolamine	*Hyoscyamus niger*, *Datura* sp., *Brugmansia* sp., *Duboisia* sp.	
**Piperidine alkaloids**	Lysine acts as precursor	Piperine	*Piper nigrum*, *P. longum*	[15,16,17]
**Quinolizidine alkaloids**		Lupinine	*Lupinus argenteus*	
		Cytisine	*Laburnum anagyroides*, *L. alpinum, Cytisus canariensis*	
**Indolizidine alkaloids**		Swainsonine	*Astragalus earlei*, *A. mollissimus*, *A. wootoni*, *A. pehuenches*, *Oxytropis lambertii*, *O. sericea*, *O. campestris*, *Swainsona luteola*, *S. greyana*, *S. galegifolia*	
**Pyridine alkaloids**	L-Aspartate acts as precursor	Nicotine	*Nicotiana tabacum*, *Nicotiana rustica*, *Duboisia hopwoodii*	[18,19]
**Pyridinone alkaloids**		Cerpegin	*Ceropegia bulbosa*, *C. juncea,*	
**Quinoline alkaloids**	Anthranillic acid acts as precursor	Skimmianine	*Skimmia japonica*, *Zanthoxylum nitidum Camptotheca acuminata*	[20,21,22]
		Camptothecin		
**Quinazoline alkaloids**		Vasicine	*Adhatoda vasica*, *Peganum harmala*	
**Xanthine alkaloid**	Adenosine (SAM cycle) acts as precursor	Theobromine	*Theobroma cacao*, *Camellia sinensis, Cola acuminate*, *Paullinia cupana*, *Ilex guayusa*	[23]
		Caffeine	*Coffea arabica*, *C. canephora*, *C. liberica*, *C. racemose*, *Theobroma cacao, Camellia sinensis*, *Cola acuminate*, *Paullinia cupana*, *Ilex guayusa*, *I. vomitoria*, *I. paraguariensis*	
**Steroid alkaloids**	Formed by the inclusion of one or two nitrogen atoms to a preformed steroid molecule	Veratridine, jervine and cyclopamine;	*Veratrum album*, *V. californicum*, *V. viride*, *Schoenocaulon officinale*	[24,25]
		Zygacine	*Toxicoscordion venenosum*, *Zigadenus glaberrimus*	
**Terpenoid** **alkaloids**	Formed by the introduction of a nitrogen atom from methylamine, ethylamine, or β-aminoethanol to terpenoidal skeletons	Secodaphniphyllate	*Daphniphyllum macropodum, D. teijsmanni, D. humile*	[26]
		Aconitine	*Aconitum napellus*, *A. variegatum*, *A. noveboracense*, *A. vulparia*, *A. delphinifolium*	
**Chromone alkaloids**	Formed by the linkage of a structure consisting of a nitrogen system to the “A” ring of chromone	Rohitukine	*Amoora rohituka*, *Dysoxylum binectariferum*, *Schumanniophyton magnificum*, *S. problematicum*	[27,28,29,30]
		Dysoline	*Dysoxylum binectariferum*	
		Cassiadinine	*Senna siamea*	
		Ficine and isoficine	*Ficus pantoniana*	
**Flavoalkaloids**		capitavine	*Buchenavia capitata*	
		Aquiledine, isoaquiledine	*Aquilegia ecalcarata*	
**Chemical Structure—Alkaloids Grouped based on Nature of Heterocyclic Ring**				
**Heterocyclic alkaloids (typical alkaloids)**	Mononuclear	Hygrine	*Erythroxylum coca, Convolvulus hanadae*	[31,32,33]
		Boldine	*Peumus boldus*, *Lindera aggregata*	
	Polynuclear	Atropine	*Atropa belladonna*, *Datura innoxia*, *D. metel, D. stramonium, Brugmansia* sp., *Hyoscyamus* sp.	
		Reserpine	*Rauvolfia serpentine*	
		Quinine	*Cinchona officinalis*	
**Non-heterocyclic alkaloids (atypical alkaloids)**	Phenylethylamine skeleton	Ephedrine	*Ephedra sinica*, *E. viridis*, *E. fragilis*, *E. distachya*, *E. ciliate*	[34,35,36]
		Capsaicin	*Capsicum frutescens*, *C. annuum*, *C. chinense*, *C. baccatum*	
	Tropolone skeleton	Colchicine	*Colchicum autumnale*, *Gloriosa superba*	
	Modified diterpenes	Paclitaxel	*Taxus baccata*, *T. brevifolia*, *T. chinensis*	
**Taxonomy—Alkaloids Grouped based on Distribution by Botanical Origin**				
**Opium alkaloids**	Present in the Papaveraceae family	Morphine, codeine, papeverine, and thebaine	*Papaver somniferum*	[37]
**Solanum alkaloids**	Present in the Solanaceae family	Solanine, tomatidine, and solasodamine	*Solanum tuberosum*, *S. lycopersicum, S. melongena*	[5]
**Daphniphyllum alkaloids**	Present in the Daphniphyllaceae family	Daphniphylline, daphilactone-B	*Daphniphyllum macropodum*	[26]
**Vinca alkaloids**	Present in the Apocynaceae family	Catharanthine leurosine, vincristine, and vinblastine	*Catharanthus roseus*	[38]
**Protoberberine alkaloids**	Present in Annonaceae Ranunculaceae, Berberidaceae, Menispermaceae families; shares the same protoberberine skeleton	Berberine	*Berberis aristata*, *B. aquifolium*, *B. heterophylla*	[9,39,40]
		Jatrorrhizine	*Enantia chlorantha*, *Thalictrum lucidumm*, *Thalictrum revolutum*	
		Palmatine	*Phellodendron amurense*, *Guatteria friesiana*	
**Ephedra alkaloids**	Present in Ephedra genus of the Ephedraceae family	Ephedrine	*Ephedra sinica*, *E. viridis*, *E. fragilis*, *E. distachya*, *E. ciliate*	[41]

**Table 2:** The biological activities of alkaloids. Numerous biological activities of alkaloids present in plants, their mechanism of action, and IC50 values are listed.

Alkaloid	Biological Activities	Mechanism of Action	IC50 Range	References
**Paclitaxel**	Antineoplastic and antimicrotubule	Suppresses microtubule dynamics by binding to β-tubulin subunits of microtubule and thereby inhibiting spindle function.	0.00126–12.3 µM	[123]
**Vincristine**	Antileukemic, antilymphoma, antineuroblastoma, and antisarcoma	Inhibits mitosis at the metaphase stage by interacting with tubulin; interferes with amino acids, cyclic AMP, glutathione metabolism, and calmodulin-dependent Ca_2_^+^-transport ATPase activity.	0.00126–1.01e + 3 µM	[123]
**Camptothecin**	Antitopoisomerase and anti-HIV	Causes DNA damage by binding to topoisomerase I and the DNA complex forming a ternary complex, stabilizing it, and preventing DNA re-ligation resulting in apoptosis.	0.00214–62.3 µM	[21]
**Rohitukine**	Anti-inflammatory, anti-fertility, anti-implantation, anti-cancer, and immuno-modulatory	Triggers apoptosis in lung cancer cells.	0.3–7.3 μM	[28]
**Strychnine**	Neurotoxic, pesticidal, and rodenticidal	Acts as an antagonist of glycine (an inhibitory neurotransmitter) and acetylcholine receptors, thereby preventing inhibitory signals and activating motor neurons in the spinal cord, resulting into spastic muscle contraction.	64–92 nM	[124]
**Ephedrine**	Promotes short-term weight loss, decreases motion sickness, possesses a cardiac stimulant, hyperglycaemic, hypertensive, bronchodilator	Indirectly stimulates the adrenergic receptor system by increasing the activity of norepinephrine at the postsynaptic α and β receptors. Acts as a CNS stimulant, due to its ability to cross the blood-brain barrier.	124 μM	[125]
**Colchicine**	Anti-gout, anti-inflammation, and treats familial Mediterranean fever	Inhibits mitosis by inhibiting microtubule polymerization; inhibits proinflammatory mechanisms and increases anti-inflammatory mediators; inhibits neutrophil motility and activity, interferes with superoxide formation, and thereby inhibits or prevents gout inflammation.	3–300 nM	[35]
**Vasicine**	Bronchodilator, mucolytic, antitussive, antibacterial, cytotoxic, abortifacient, and uterotonic	Acts as an acetylcholinesterase inhibitor and a butyrylcholinesterase inhibitor.	125 μM	[126,127]
**Reserpine**	Anti-hypertensive and anti-psychotic	Interferes with the sequestering of neurotransmitters into storage vesicles located in the presynaptic neuron by inhibiting their ATP/Mg^2+^ pump, causing a reduction in catecholamines, thereby causing antihypertensive effects.	1.7–2.8 μM	[128]
**Ajmalicin**	Anti-hypertensive	Acts as α1-adrenergic receptor antagonist and shows hypotensive effects.	3.5–5.44 μM	[11]
**Tetrandrine**	Anti-inflammatory, immunologic, anti-allergenic, and anti-tumour; used for treating Ebola virus infection in mice	Acts as a calcium-channel blocker, inhibits the degranulation of mast cells.	11.3 μM	[129]
**Morphine**	Analgesic and CNS stimulant	Acts as agonists for mu and kappa opioid receptors, on the ventral tegmental area of the brain; agonist of the delta-opioid receptor in the nucleus accumbens and activates the morphine reward pathway.	1–8.8 mM	[37]
**Codeine**	Analgesic, antidiarrheal, and antitussive	Acts as agonist for mu opioid receptors involved in the transmission of pain throughout the body and central nervous system.	60 μM	[130]
**Papaverine**	Vasodilatory and antispasmodic	Shows direct vasodilating action on cerebral blood vessels, increases cerebral blood flow and decreases cerebral vascular resistance.	2–37 μM	[6]
**Berberine**	Antimicrobial, antitumor, anti-hyperglycemic, antimalarial, and anti-inflammation; Alzheimer’s disease treatment	Lowers cholesterol through LDL-receptor-mediated liver LDL cholesterol clearance, promotes LDL-receptor expression through the proprotein convertase subtilisin/kexin type 9 (PCSK9)-LDL-receptor pathway.	0.1–25 µM	[131]
**Scopolamine**	Depressant action on sympathetic nervous system; possesses mydriatic, spasmolytic, and local anesthetic effects; treats motion sickness, postoperative nausea, and vomiting	Acts as a non-selective competitive inhibitor of M1-M5 mAChRs (G-protein-coupled muscarinic acetylcholine receptors), shows anticholinergic effect, and alters signalling through CNS associated with vomiting.	928 µM	[72]
**Piperine**	Presents hemo-preventive, anti-carcinogenic, antioxidant, anti-inflammatory, anticarcinogenic, stimulatory, hepatoprotective, antihyperlipidemia, anti-asthmatic activities; gastro-intestinal stimulant, and appetite stimulant	Affects the plasma concentrations of P-glycoprotein in the (P-gp)-mediated transport of drugs and metabolizes enzyme CYP3A4 substrates in humans; lowers endogenous UDP-glucuronic acid contents and inhibits transferase activity, thereby modifying the rate of glucuronidation.	1–34 μM	[132]
**Lupinine**	Insecticidal	Reversible inhibitor of acetylcholinesterases; possesses a binding affinity for muscarinic and nicotinic acetylcholine receptors.	712 μM	[133]
**Swainsonine**	Chemotherapeutic	Acts as a golgi α-mannosidase II inhibitor	34 nM	[134]
**Skimmianine**	Analgesic, antispastic, sedative, and anti-inflammatory	Suppresses TNF-α and IL-6 gene transcription, inhibits the production of NO, prostaglandin E2, and superoxide anions.	8.6 μg/mL	[20]
**Theobromine**	Antitumor, bronchorelaxater, and antitussive	Acts as antagonist to adenosine-receptors within the plasma membrane of virtually every cell, which further promotes neurotransmitter release.	2500 µM	[135]
**Caffeine**	Autonomous nervous system stimulant, anti-inflammation; improves cognitive performance	Inhibits the activity of nucleotide phosphodiesterase enzymes, regulates calcium handling in cells, and participates in adenosine receptor antagonism, stimulating inotropic effects in the heart.	500–1000 μM	[136]
**Nicotine**	Antiherbivore, insecticide, teratogenic, addictive, stimulant, and anxiolytic effects; treatment of nicotine dependence	Acts as an agonist/antagonist of certain nicotinic acetylcholine receptors, binding with receptors leading to depolarization, activating voltage-gated calcium channels.	0.5–20 nM	[137,138]
**Veratridine**	Inhibitor of sodium channel inactivation and neurotoxic	Depolarizes cells by affecting sodium channels, can activate Nav 1.8 along with additional Nav channels; enhances protein tyrosine phosphorylation; can turn the membrane potential to a more positive one and can also modify the effect of progesterone on (a_2_+)i and sperm membrane potential.	27–84 µM	[139]
**Aconitine**	Analgesic, blood coagulant, anti-inflammatory, cardiotoxic, and neurotoxic	Interacts with voltage-dependent sodium-ion channels, binds to the channel at the neurotoxin binding site 2 on the α-subunit, suppressing the conformational change in the sodium-ion channel from an active state to an inactive state.	10–20 µM	[140]
**Hygrine**	Sedative, hypnotic laxative, and diuretics	Not known.	Not reported	[31]
**Boldine**	Antioxidant, antipyretic, anti-inflammation, hepatoprotectant, cytoprotectant, and neuroprotectant	Acts as an α-adrenergic antagonist in vascular tissues; it can cross the blood-brain barrier exhibiting neuroprotective effects.	8.5 µM	[141]
**Atropine**	Anticholinergic, antispasmodic, and antimuscarinic	Binds and inhibits muscarinic acetylcholine receptors, producing anticholinergic effects.	2–55 µM	[32]
**Capsaicin**	Anti-obesity, antifungal action, and chemical irritant; treating peripheral neuropathy, psoriasis, and non-allergic rhinitis	Induces a topical hypersensitivity reaction on the skin by carrying out the “defunctionalization” of nociceptor fibers. Pain mechanism is due to temporary loss of membrane potential, inability to transport neurotrophic factors, and the reversible retraction of epidermal and dermal nerve fiber terminals.	50 μM	[142]
**α-Solanine**	Antiallergic, anti-inflammation, antipyretic, and anti-carcinogen; treating gastrointestinal and neurological disorders	Inhibits cholinesterase activity, disrupts cell membranes; opens the potassium channels of the mitochondria increasing their membrane potential, followed by the transport of Ca_2_+ from mitochondria into the cytoplasm leading to the an increased concentration of Ca_2_+ in the cytoplasm triggering cell damage and apoptosis.	32.18 μM	[143]
**α-Tomatine**	Anti-leukemia, fungicide, antimicrobial, and insecticide	Causes the disruption of cellular membranes and the inhibition of acetylcholinesterase; stimulates the immune system by participation in a sequence of respiratory burst destroying bacteria.	7–10 μM	[144]
**Jatrorrhizine**	Antibacterial and antifungal	Blocks α-1 and α-2 adrenoreceptors and monoamine oxidase A and B.	4–62 μM	[98]
**Palmatine**	Antimicrobial, hypoglycemic, antiarrhythmic, and antioxidant	Intercalates with nucleic acids; induces apoptosis; inhibits proliferation.	0.07–22 µM	[145]
**Quinine**	Antimalaria, mild antipyretic, and analgesic	Interferes with a parasite’s ability to break down and digest hemoglobin, leading to starvation in parasites.	13.4 µM	[146]
**Cytisine**	Teratogenic	Partial agonist of α4-β2 nicotinic acetylcholine receptors; causes a reduction in the effects of nicotine on dopamine release in the mesolimbic system when given alone, while simultaneously attenuates nicotine withdrawal symptoms accompanying cessation attempts.	27.3 nM	[147]

**Table 3:** Commercial applications of plant alkaloids. Approved formulations of alkaloids and their potential applications have been listed. Information about the formulations has been retrieved from web sources.

Application	Constituent Alkaloid	Formulation Names
Chemotherapy	Paclitaxel	Taxol^®^, Taxotere^®^;
	Vinorelbine prepared from vindoline and catharanthine	Navelbine^®^;
	Vinblastine	Velban^®^;
	Vincristine	Vincasar Pfs^®^, Oncovin^®^;
	Camptothecin	Camptosar^®^
Gout treatment	Colchicin	Colcrys, Mitigare, Gloperba
Respiratory ailments treatment	Vasicine	Ayusas Adulsa Cough syrup;
	Codeine	Ambenyl^®^, Calcidrine, Neo AC cough syrup;
	Capsaicin	Nasol Nasal spray^TM^
Hypertension treatment	Reserpine	Diupres-250, Diupres-500, Regroton^®^, Demi-Regroton;
	Ajmalicine	Isosarpan, Iskedyl, Isquebral, Duxil, Duxor, Saltucin Co, Salvalion, Sarpan;
Anesthetic premedication, toxicity antidotes	Atropine	Atropen
Antimuscarinic agents	Atropine	Isopto Atropine, Vistatropine Eye Drops
Analgesic agents	Morphine	Kadian, Kadian ER, Morphabond, Oramorph SR,
		Roxanol;
	Codeine	Emperine 3;
	Capsaicin	Capsitop O Roll ON, Zostrix, Capzasin-HP, Axsain, Rid-A-Pain, Salonpas Hot, Medigrip Capsicum Plaster
Cardiac ailment treatment	Papaverin	Pavabid^®^ (Marion), Papaver^TM^
Malaria treatment	Quinine	Qualaquin,
Scalp repairment	Capsaicin	Thermascalp
Nutritional supplement	Ephedrine	ECA Stack;
	Berberine	Berberta, Myobery Tablet, Berberine Glucose Support, Berbitol Tablet;
	Piperine	Superb^TM^ Qp, Rhodiola, Dezcumin
	Vincamine	Oxybral SR, Brain Ox, Vincabral SR
Smoking cessation	Cytosine	Tabex
	Nicotine	Nicotex Nicotine Gum,
		STOP-NIC Nicotine Gum, Nixit-Nicotine Gum
Pesticide	Strychnine	Boomer-Rid, Certox, Dog-button, Dolco mouse Ceral, Stricnina, Mole death, Mouse-nots, Strychnos

## Data Availability

Not applicable.

## References

[1] Pott D.M., Osorio S., Vallarino J.G. (2019). From central to specialized metabolism: An overview of some secondary compounds derived from the primary metabolism for their role in conferring nutritional and organoleptic characteristics to fruit. Front. Plant Sci..

[2] Kuramoto M., Arimoto H., Uemura D. (2004). Bioactive alkaloids from the sea: A review. Mar. Drugs.

[3] Braekman J.C., Daloze D., Pasteels J.M., Roberts M.F., Wink M. (1998). Alkaloids in animals. Alkaloids: Biochemistry, ecology, and medicinal applications.

[4] Hartmann T. (2007). From waste products to ecochemicals: Fifty years research of plant secondary metabolism. Phytochemistry.

[5] Cárdenas P.D., Sonawane P.D., Heinig U., Heinig U., Jozwiak A., Panda S., Abebie B., Kazachkova Y., Pliner M., Unger T. (2019). Pathways to defense metabolites and evading fruit bitterness in genus *Solanum* evolved through 2-oxoglutarate-dependent dioxygenases. Nat. Commun..

[6] Beaudoin G.A.W., Facchini P.J. (2014). Benzylisoquinoline alkaloid biosynthesis in opium poppy. Planta.

[7] Matsuura H.N., Fett-Neto A.G., Gopalakrishnakone P., Carlini C., Ligabue-Braun R. (2017). Plant alkaloids: Main features, toxicity and mechanisms of action. Plant Toxins.

[8] Dey P., Kundu A., Kumar A., Gupta M., Lee B.M., Bhakta T., Dash S., Kim H.S. (2020). Analysis of alkaloids (indole alkaloids, isoquinoline alkaloids, tropane alkaloids). Recent Adv. Nat. Prod. Anal..

[9] Zhang Y.-T., Yu Y.-Q., Yan X.-X., Wang W.-J., Tian X.-T., Wang L., Zhu W.-L., Gong L.-K., Pan G.-Y. (2019). Different structures of berberine and five other protoberberine alkaloids that affect P-glycoprotein-mediated efflux capacity. Acta. Pharmacol. Sin..

[10] Kurz W.G., Chatson K.B., Constabel F., Kutney J.P., Choi L.S.L., Kolodziejczyk P., Sleigh S.K., Stuart K.L., Worth B.R. (1981). Alkaloid production in *Catharanthus roseus* cell cultures VIII1. Planta Med..

[11] León F., Habib E., Adkins J.E., Furr E.B., McCurdy C.R., Cutler S.J. (2009). Phytochemical characterization of the leaves of *Mitragyna speciosa* grown in U.S.A. Nat. Prod. Commun..

[12] Bonjoch J., Solé D. (2000). Synthesis of Strychnine. Chem. Rev..

[13] Smith L.W., Culvenor C.C.J. (1981). Plant sources of hepatotoxic pyrrolizidine alkaloids. J. Nat. Prod..

[14] Moharrami F., Hosseini B., Sharafi A., Farjaminezhad M. (2017). Enhanced production of hyoscyamine and scopolamine from genetically transformed root culture of *Hyoscyamus reticulatus* L. elicited by iron oxide nanoparticles. In Vitro Cell Dev. Biol. Plant.

[15] Chopra B., Dhingra A.K., Kapoor R.P., Prasad D.N. (2016). Piperine and its various physicochemical and biological aspects: A review. Open Chem. J..

[16] Wink M., Meißner C., Witte L. (1995). Patterns of quinolizidine alkaloids in 56 species of the genus Lupinus. Phytochemistry.

[17] Cook D., Gardner D.R., Pfister J.A. (2014). Swainsonine-containing plants and their relationship to endophytic fungi. J. Agric. Food Chem..

[18] Fagerström K. (2014). Nicotine: Pharmacology, toxicity and therapeutic use. J. Smok. Cessat..

[19] Gharat S.A., Shinde B.A., Mule R.D., Punekar S.A., Dholakia B.B., Jayaramaiah R.H., Ramaswamy G., Giri A.P. (2020). High-throughput metabolomic and transcriptomic analyses vet the potential route of cerpegin biosynthesis in two varieties of *Ceropegia bulbosa Roxb*. Planta.

[20] Yang Z.-D., Zhang D.-B., Ren J., Yang M.-J. (2012). Skimmianine, a furoquinoline alkaloid from *Zanthoxylum nitidum* as a potential acetylcholinesterase inhibitor. Med. Chem. Res..

[21] Li Y.-Y., Chen S.-W., Yang L.-M., Wang R.-R., Pang W., Zheng Y.-T. (2010). The Anti-HIV actions of 7- and 10-substituted camptothecins. Molecules.

[22] Bhambhani S., Karwasara V.S., Dixit V.K., Banerjee S. (2012). Enhanced production of vasicine in *Adhatoda vasica* (L.) Nees. cell culture by elicitation. Acta Physiol. Plant.

[23] Caballero B., Finglas P., Toldra F. (2015). Encyclopedia of food and health.

[24] Chandler C.M., McDougal O.M. (2014). Medicinal history of north american veratrum. Phytochem. Rev..

[25] Schwartz F.C., FNA Editorial Committee (2003). “Zigadenus glaberrimus Michaux, Fl. Bor.-Amer. 1: 214, plate 22. 1803”. Magnoliophyta: Liliales and Orchidales. Flora of North America. 26.

[26] Dong M., Zhang M.L., Shi Q.W., Gu Y.C., Kiyota H. (2009). The daphniphyllum alkaloids. Curr. Org. Chem..

[27] Khadem S., Marles R.J. (2012). Chromone and flavonoid alkaloids: Occurrence and bioactivity. Molecules.

[28] Kumar V., Guru S.K., Jain S.K., Joshi P., Gandhi S.G., Bharate S.B., Bhushan S., Bharate S.S., Vishwakarma R.A. (2016). A chromatography-free isolation of rohitukine from leaves of *Dysoxylum binectariferum*: Evaluation for *in vitro* cytotoxicity, Cdk inhibition and physicochemical properties. Bioorg. Med. Chem. Lett..

[29] Coffin A., Ready J.M. (2019). Selective Synthesis of (+)-Dysoline. Org. Lett..

[30] Biswas K., Mallik H. (1986). Cassiadinine, a chromone alkaloid and (+)-6-hydroxy-mellein, a dihydroisocoumarin from *Cassia siamea*. Phytochemistry.

[31] Talapatra S.K., Talapatra B. (2015). Hygrine, hygroline, and cuscohygrine (ornithine-derived alkaloids). Chemistry of Plant Natural Products.

[32] Kohnen-Johannsen K.L., Kayser O. (2019). Tropane alkaloids: Chemistry, pharmacology, biosynthesis and production. Molecules.

[33] Lobay D. (2015). Rauwolfia in the treatment of hypertension. Integr. Med. Encinitas.

[34] Abourashed E.A., El-Alfy A.T., Khan I.A., Walker L. (2003). Ephedra in perspective—A current review. Phytother. Res..

[35] Nett R.S., Lau W., Sattely E.S. (2020). Discovery and engineering of colchicine alkaloid biosynthesis. Nature.

[36] Croteau R., Ketchum R.E., Long R.M., Kaspera R., Wildung M.R. (2006). Taxol biosynthesis and molecular genetics. Phytochem. Rev..

[37] Carlin M.G., Dean J.R., Ames J.M. (2020). Opium alkaloids in harvested and thermally processed poppy seeds. Front. Chem..

[38] Williams D., Qu Y., Simionescu R., De Luca V. (2019). The assembly of (+)-vincadifformine- and (-)-tabersonine-derived monoterpenoid indole alkaloids in *Catharanthus roseus* involves separate branch pathways. Plant J..

[39] Virtanen P., Lassila V., Njimi T., Mengata D.E. (1988). Natural protoberberine alkaloids from *Enantia chlorantha,* palmatine, columbamine and jatrorrhizine for thioacetamide-traumatized rat liver. Acta. Anat..

[40] Tarabasz D., Kukula-Koch W. (2020). Palmatine: A review of pharmacological properties and pharmacokinetics. Phytother Res..

[41] Elhadef K., Smaoui S., Fourati M., Ben Hlima H., Chakchouk Mtibaa A., Sellem I., Ennouri K., Mellouli L. (2020). A review on worldwide Ephedra history and story: From fossils to natural products mass spectroscopy characterization and biopharmacotherapy potential. Evid. Based Complementary Altern. Med..

[42] Lichman B.R. (2021). The scaffold-forming steps of plant alkaloid biosynthesis. Nat. Prod. Rep..

[43] Krayer O., Meilman E., Gross F. (1977). Veratrum alkaloids with antihypertensive activity. Antihypertensive Agents.

[44] O’Connor S.E., Maresh J.J. (2006). Chemistry and biology of monoterpene indole alkaloid biosynthesis. Nat. Prod. Rep..

[45] Neag M.A., Mocan A., Echeverría J., Pop R.M., Bocsan C.I., Crişan G., Buzoianu A.D. (2018). Berberine: Botanical occurrence, traditional uses, extraction methods, and relevance in cardiovascular, metabolic, hepatic, and renal disorders. Front. Pharmacol..

[46] González-Juárez D.E., Escobedo-Moratilla A., Flores J., Hidalgo-Figueroa S., Martínez-Tagüeña N., Morales-Jiménez J., Muñiz-Ramírez A., Pastor-Palacios G., Pérez-Miranda S., Ramírez-Hernández A. (2020). A review of the *Ephedra* genus: Distribution, ecology, ethnobotany, phytochemistry and pharmacological properties. Molecules.

[47] Kumar V., Satyanarayana K., Ramakrishna A., Chandrashekar A., Ravishankar G. (2007). Evidence for localization of N-methyltransferase (NMT) of caffeine biosynthetic pathway in vacuolar surface of *Coffea canephora* endosperm elucidated through localization of GUS reporter gene driven by NMT promoter. Curr. Sci..

[48] Facchini P., Morris J. (2019). Molecular origins of functional diversity in benzylisoquinoline alkaloid methyltransferases. Front. Plant Sci..

[49] Agarwal P., Pathak S., Kumar R.S., Dhar Y.V., Pandey A., Shukla S., Trivedi P.K. (2019). 3′*O*-Methyltransferase, *Ps3′OMT*, from opium poppy: Involvement in papaverine biosynthesis. Plant Cell Rep..

[50] Menéndez-Perdomo I.M., Facchini P.J. (2018). Benzylisoquinoline Alkaloids biosynthesis in Sacred Lotus. Molecules.

[51] Menéndez-Perdomo I.M., Facchini P.J. (2020). Isolation and characterization of two O-methyltransferases involved in benzylisoquinoline alkaloid biosynthesis in sacred lotus (*Nelumbo nucifera*). J. Biol. Chem..

[52] Wink M. (1984). N-Methylation of quinolizidine alkaloids: An S-adenosyl-L-methionine: Cytisine N-methyltransferase from Laburnum anagyroides plants and cell cultures of *L. alpinum* and *Cytisus canariensis*. Planta.

[53] Jozwiak A., Sonawane P.D., Panda S., Garagounis C., Papadopoulou K.K., Abebie B., Massalha H., Almekias-Siegl E., Scherf T., Aharoni A. (2020). Plant terpenoid metabolism co-opts a component of the cell wall biosynthesis machinery. Nat. Chem. Biol..

[54] Mylona P., Owatworakit A., Papadopoulou K., Jenner H., Qin B., Findlay K., Hill L., Qi X., Bakht S., Melton R. (2008). Sad3 and sad4 are required for saponin biosynthesis and root development in oat. Plant Cell.

[55] Naoumkina M.A., Zhao Q., Gallego-Giraldo L., Dai X., Zhao P.X., Dixon R.A. (2010). Genome-wide analysis of phenylpropanoid defence pathways. Mol. Plant Pathol..

[56] McCue K.F., Allen P.V., Shepherd L.V., Blake A., Maccree M.M., Rockhold D.R., Novy R., Stewart D., Davies H., Belknap W. (2007). Potato glycosterol rhamnosyltransferase, the terminal step in triose sidechain biosynthesis. Phytochemistry.

[57] Ginzberg I., Tokuhisa J.G., Veilleux R.E. (2009). Potato steroidal glycoalkaloids: Biosynthesis and genetic manipulation. Potato Res..

[58] Itkin M., Rogachev I., Alkan N., Rosenberg T., Malitsky S., Masini L., Meir S., Iijima Y., Aoki K., de Vos R. (2011). *GLYCOALKALOID METABOLISM1* is required for steroidal alkaloid glycosylation and prevention of phytotoxicity in tomato. Plant Cell.

[59] Itkin M., Heinig U., Tzfadia O., Bhide A.J., Shinde B., Cardenas P.D., Bocobza S.E., Unger T., Malitsky S., Finkers R. (2013). Biosynthesis of antinutritional alkaloids in solanaceous crops is mediated by clustered genes. Science.

[60] Zhang Y.J., Xie K.B., Liu A.J., Chen R.D., Chen D.W., Yang L., Dai J.G. (2019). Enzymatic biosynthesis of benzylisoquinoline alkaloid glycosides via promiscuous glycosyltransferases from *Carthamus tinctorius*. Chin. Chem. Lett..

[61] Schmidt G.W., Jirschitzka J., Porta T., Reichelt M., Luck K., Torre J.C., Dolke F., Varesio E., Hopfgartner G., Gershenzon J. (2015). The last step in cocaine biosynthesis is catalyzed by a BAHD acyltransferase. Plant Physiol..

[62] Yu X.H., Chen M.H., Liu C.J. (2008). Nucleocytoplasmic-localized acyltransferases catalyze the malonylation of 7-O-glycosidic (iso)flavones in *Medicago truncatula*. Plant J..

[63] Panikashvili D., Shi J.X., Schreiber L., Aharoni A. (2009). The arabidopsis DCR encoding a soluble BAHD acyltransferase is required for cutin polyester formation and seed hydration properties. Plant Physiol..

[64] Mugford S.T., Louveau T., Melton R., Qi X., Bakht S., Hill L., Tsurushima T., Honkanen S., Rosser S.J., Lomonossoff G.P. (2013). Modularity of plant metabolic gene clusters: A trio of linked genes that are collectively required for acylation of triterpenes in oat. Plant Cell.

[65] Grothe T., Lenz R., Kutchan T.M. (2001). Molecular characterization of the salutaridinol 7-O-acetyltransferase involved in morphine biosynthesis in opium poppy *P. somniferum*. J. Biol. Chem..

[66] Carqueijeiro I., Dugé de Bernonville T., Lanoue A., Dang T.T., Teijaro C.N., Paetz C., Billet K., Mosquera A., Oudin A., Besseau S. (2018). A BAHD acyltransferase catalyzing 19-*O*-acetylation of tabersonine derivatives in roots of *Catharanthus roseus* enables combinatorial synthesis of monoterpene indole alkaloids. Plant J..

[67] Qiu F., Zeng J., Wang J., Huang J.P., Zhou W., Yang C., Lan X., Chen M., Huang S.X., Kai G. (2020). Functional genomics analysis reveals two novel genes required for littorine biosynthesis. New Phytol..

[68] Christ B., Xu C., Xu M., Li F.S., Wada N., Mitchell A.J., Han X.-L., Wen M.-L., Fujita M., Weng J.-K. (2019). Repeated evolution of cytochrome P450-mediated spiroketal steroid biosynthesis in plants. Nat. Commun..

[69] Nelson D.R., Schuler M.A. (2013). Cytochrome P450 genes from the sacred lotus genome. Trop. Plant Biol..

[70] Nishimura K., Horii S., Tanahashi T., Sugimoto Y., Yamada J. (2013). Synthesis and pharmacological activity of alkaloids from embryo of lotus, *Nelumbo nucifera*. Chem. Pharm. Bull..

[71] Li R., Reed D.W., Liu E., Nowak J., Pelcher L.E., Page J.E., Covello P.S. (2006). Functional genomic analysis of alkaloid biosynthesis in *Hyoscyamus niger* reveals a cytochrome P450 involved in littorine rearrangement. Chem. Biol..

[72] Hashimoto T., Matsuda J., Yamada Y. (1993). Two-step epoxidation of hyoscyamine to scopolamine is catalyzed by bifunctional hyoscyamine-6 -hydroxylase. FEBS Lett..

[73] Hagel J.M., Facchini P.J. (2013). Benzylisoquinoline alkaloid metabolism: A century of discovery and a brave new world. Plant Cell Physiol..

[74] Farrow S.C., Hagel J.M., Beaudoin G.A., Burns D.C., Facchini P.J. (2015). Stereochemical inversion of (S)-reticuline by a cytochrome P450 fusion in opium poppy. Nat. Chem. Biol..

[75] Jirschitzka J., Schmidt G.W., Reichelt M., Schneider B., Gershenzon J., D’Auria J.C. (2012). Plant tropane alkaloid biosynthesis evolved independently in the Solanaceae and Erythroxylaceae. Proc. Natl. Acad. Sci. USA.

[76] Kilgore M.B., Holland C.K., Jez J.M., Kutchan T.M. (2016). Identification of a Noroxomaritidine Reductase with Amaryllidaceae alkaloid biosynthesis related activities. J. Biol. Chem..

[77] Parage C., Foureau E., Kellner F., Burlat V., Mahroug S., Lanoue A., Dugé de Bernonville T., Londono M.A., Carqueijeiro I., Oudin A. (2016). Class II cytochrome P450 reductase governs the biosynthesis of alkaloids. Plant Physiol..

[78] Madyastha K.M., Coscia C.J. (1979). Detergent-solubilized NADPH cytochrome c(P-450) reductase from the higher plant, *Catharanthus roseus*: Purification and characterization. J. Biol. Chem..

[79] Meijer A.H., Lopes Cardoso M.I., Voskuilen J.T., de Waal A., Verpoorte R., Hoge J.H.C. (1993). Isolation and characterization of a cDNA clone from *Catharanthus roseus* encoding NADPH: Cytochrome P-450 reductase, an enzyme essential for reactions catalysed by cytochrome P-450 monooxygenases in plants. Plant J..

[80] Sun L., Ruppert M., Sheludko Y., Warzecha H., Zhao Y., Stöckigt J. (2008). Purification, cloning, functional expression and characterization of perakine reductase: The first example from the AKR enzyme family, extending the alkaloidal network of the plant *Rauvolfia*. Plant Mol. Biol..

[81] Wu C., Yang K., Liu Q., Wakui M., Jin G.Z., Zhen X., Wu J. (2010). Tetrahydroberberine blocks ATP-sensitive potassium channels in dopamine neurons acutely-dissociated from rat substantia nigra pars compacta. Neuropharmacology.

[82] Gesell A., Rolf M., Ziegler J., Diaz Chavez M.L., Huang F.-C., Kutchan T.M. (2009). CYP719B1 Is salutaridine synthase, the C-C phenol-coupling enzyme of morphine biosynthesis in Opium Poppy. J. Biol. Chem..

[83] Matsuoka E., Matsubara T., Takahashi I., Murano H., Hara M. (2016). The isoquinoline alkaloid sanguinarine which inhibits chaperone activity enhances the production of heat shock proteins in *Arabidopsis*. Plant Biotechnol.

[84] Wangchuk P., Keller P.A., Pyne S.G., Willis A.C., Kamchonwongpaisan S. (2012). Antimalarial alkaloids from a Bhutanese traditional medicinal plant *Corydalis dubia*. J. Ethnopharmacol..

[85] Saeed S.A., Gilani A.H., Majoo R.U., Shah B.H. (1997). Anti-thrombotic and anti-inflammatory activities of protopine. Pharmacol. Res..

[86] Qu Y., Easson M.L., Froese J., Simionescu R., Hudlicky T., De Luca V. (2015). Completion of the seven-step pathway from tabersonine to the anticancer drug precursor vindoline and its assembly in yeast. Proc. Natl. Acad. Sci. USA.

[87] Laflamme P., St-Pierre B., De Luca V. (2001). Molecular and biochemical analysis of a Madagascar Periwinkle root-specific minovincinine-19-hydroxy-O-acetyltransferase. Plant Physiol..

[88] Stander E.A., Sepúlveda L.J., Dugé de Bernonville T., Carqueijeiro I., Koudounas K., Lemos Cruz P., Besseau S., Lanoue A., Papon N., Giglioli-Guivarc’h N. (2020). Identifying genes involved in alkaloid biosynthesis in vinca minor through transcriptomics and gene co-expression analysis. Biomolecules.

[89] Abouzeid S., Beutling U., Surup F., Abdel Bar F.M., Amer M.M., Badria F.A., Yahyazadeh M., Brönstrup M., Selmar D. (2017). Treatment of vinca minor Leaves with methyl jasmonate extensively alters the pattern and composition of indole alkaloids. J. Nat. Prod..

[90] Kellner F., Geu-Flores F., Sherden N.H., Brown S., Foureau E., Courdavault V., O’Connor S.E. (2015). Discovery of a P450-catalyzed step in vindoline biosynthesis: A link between the aspidosperma and eburnamine alkaloids. Chem. Commun..

[91] Ettefagh K.A., Burns J.T., Junio H.A., Kaatz G.W., Cech N.B. (2011). Goldenseal (*Hydrastis Canadensis* L.) extracts synergistically enhance the antibacterial activity of berberine *via* efflux pump inhibition. J. Planta Med..

[92] Yohannes A., Eyalarasan K., Imam, Eyob L., Ghebrengus E., Weldemariam L., Yohannes T., Yemane A. (2018). Antibacterial and antifungal activities of easily grown Eritrean black pepper. Int. J. Eng. Res. Technol..

[93] Cushnie T.P., Cushnie B., Lamb A.J. (2014). Alkaloids: An overview of their antibacterial, antibiotic-enhancing and antivirulence activities. Int. J. Antimicrob. Agents.

[94] Simons V., Morrissey J.P., Latijnhouwers M., Csukai M., Cleaver A., Yarrow C., Osbourn A. (2006). Dual effects of plant steroidal alkaloids on *Saccharomyces cerevisiae*. Antimicrob. Agents Chemother..

[95] Cretton S., Dorsaz S., Azzollini A., Favre-Godal Q., Marcourt L., Ebrahimi S.N., Voinesco F., Michellod E., Sanglard D., Gindro K. (2016). Antifungal quinoline alkaloids from *Waltheria indica*. J. Nat. Prod..

[96] Singh N., Azmi S., Maurya S., Singh U., Jha R., Pandey V. (2003). Two plant alkaloids isolated from *Corydalis longipes* as potential antifungal agents. Folia Microbiol..

[97] Morteza-Semnani K., Amin G., Shidfar M., Hadizadeh H., Shafiee A. (2003). Antifungal activity of the methanolic extract and alkaloids of *Glaucium oxylobum*. Fitoterapia.

[98] Thawabteh A., Juma S., Bader M., Karaman D., Scrano L., Bufo S.A., Karaman R. (2019). The biological activity of natural alkaloids against herbivores, cancerous cells and pathogens. Toxins.

[99] Kortbeek R.W.J., van der Gragt M., Bleeker P.M. (2019). Endogenous plant metabolites against insects. Eur. J. Plant Pathol..

[100] Mithöfer A., Boland W. (2012). Plant defense against herbivores: Chemical aspects. Annu. Rev. Plant Biol..

[101] Pérez S., Taroška I. (2014). Carbohydrate–protein interactions: Molecular modeling insights. Advances in Carbohydrate Chemistry and Biochemistry, Horton, D, Ed..

[102] War A.R., Buhroo A.A., Hussain B., Ahmad T., Nair R.M., Sharma H.C., Merillon J.M., Ramawat K. (2019). Plant defense and insect adaptation with reference to secondary metabolites. Co-Evolution of Secondary Metabolites. Reference Series in Phytochemistry.

[103] Siddiqui Z.S., Zaman A.U. (2005). Effects of capsicum leachates on germination, seedling growth and chlorophyll accumulation in *Vigna radiata* (L.) Wilczek seedlings. Pak. J. Bot..

[104] Christiansen J.L., Jornsgard B., Buskov S., Olsen C.E. (1997). Effect of drought stress on content and composition of seed alkaloids in narrow-leafed lupin, *Lupinus angustifolius* L.. Eur J. Agron..

[105] Szabo B., Tyihak E., Szabo L.G., Botz L. (2003). Mycotoxin and drought stress induced change of alkaloid content of *Papaver somniferum* plantlets. Acta Bot. Hung..

[106] Alhaithloul H.A., Soliman M.H., Ameta K.L., El-Esawi M.A., Elkelish A. (2020). Changes in ecophysiology, osmolytes, and secondary metabolites of the medicinal plants of *Mentha piperita* and *Catharanthus roseus* subjected to drought and heat stress. Biomolecules.

[107] Brachet J., Cosson L. (1986). Changes in the total alkaloid content of *Datura innoxia* Mill. subjected to salt stress. J. Exp. Bot..

[108] Mishra M.R., Srivastava R.K., Akhtar N. (2019). Abiotic stresses of salinity and water to enhance alkaloids production in cell suspension culture of *Catharanthus roseus*. Glob. J. Bio-Sci. Biotechnol..

[109] Wang J.Y., Liu Z.P. (2010). Alkaloid accumulation in *Catharanthus roseus* increases with addition of seawater salts to the nutrient solution. Pedosphere.

[110] Misra N., Gupta A.K. (2006). Effect of salinity and different nitrogen sources on the activity of antioxidant enzymes and indole alkaloid content in *Catharanthus roseus* seedlings. J. Plant Physiol..

[111] Binder B.Y., Peebles C.A., Shanks J.V., San K.Y. (2009). The effects of UV-B stress on production of terpenoid indole-alkaloids in *Catharanthus roseus* hairy roots. Biotechnol. Prog..

[112] Bienaime C., Melin A., Bensaddek L., Jacques A., Nava-Saucedo E., Baltora-Rosset E. (2015). Effects of plant growth regulators on cell growth and alkaloids production by cell cultures of *Lycopodiella inundata*. Plant Cell Tissue Organ. Cult..

[113] Raj D., Kokotkiewicz A., Drys A., Luczkiewicz M. (2015). Effect of plant growth regulators on the accumulation of indolizidine alkaloids in *Securinega suffruticosa* callus cultures. Plant Cell Tissue Organ. Cult..

[114] Li W., Shao Y., Hu L., Zhang X., Chen Y., Tong L., Li C., Shen X., Ding J. (2007). BM6, a new semi-synthetic *Vinca* alkaloid, exhibits its potent in vivo anti-tumor activities via its high binding affinity for tubulin and improved pharmacokinetic profiles. Cancer Biol. Ther..

[115] Gerullis H., Wawroschek F., Kohne C.-H., Ecke T. (2016). Vinflunine in the treatment of advanced urothelial cancer: Clinical evidence and experience. Ther. Adv. Urol..

[116] Lu J.J., Bao J.L., Chen X.P., Huang M., Wang Y.T. (2012). Alkaloids isolated from natural herbs as the anticancer agents. Evid. Based Complementary Altern. Med..

[117] Wang Y., Liu Y., Du X., Ma H., Yao J. (2020). The anti-cancer mechanisms of berberine: A review. Cancer Manag. Res..

[118] Shyu K.G., Lin S., Lee C.C., Chen E., Lin L.C., Wang B.W., Tsai S.C. (2006). Evodiamine inhibits *in vitro* angiogenesis: Implication for antitumorgenicity. Life Sci..

[119] Rather R.A., Bhagat M. (2018). Cancer chemoprevention and piperine: Molecular mechanisms and therapeutic opportunities. Front. Cell Dev. Biol..

[120] Slobodnick A., Shah B., Pillinger M.H., Krasnokutsky S. (2015). Colchicine: Old and new. Am. J. Med..

[121] Yang L.P.H. (2010). Oral colchicine (Colcrys®): In the treatment and prophylaxis of gout. Drugs.

[122] Bhattacharyya B., Panda D., Gupta S., Banerjee M. (2008). Anti-mitotic activity of colchicine and the structural basis for its interaction with tubulin. Med. Res. Rev..

[123] Isah T. (2016). Anticancer alkaloids from trees: Development into drugs. Pharmacogn. Rev..

[124] Otter J., D’Orazio J.L. Strychnine toxicity. In StatPearls [Internet]. Treasure Island (FL): StatPearls Publishing. https://www.ncbi.nlm.nih.gov/books/NBK459306/.

[125] Cruz A., Padilla-Martínez I.I., Bautista-Ramírez M.E., Vasil G., Atanas P. (2017). Synthesis, structure and biological activity of ephedra heterocycles. Alkaloids—Alternatives in Synthesis, Modification and Application.

[126] Liu W., Wang Y., He D.D., Li S.P., Zhu Y.D., Jiang B., Cheng X.M., Wang Z., Wang C.H. (2015). Antitussive, expectorant, and bronchodilating effects of quinazoline alkaloids (±)-vasicine, deoxyvasicine, and (±)-vasicinone from aerial parts of *Peganum harmala* L.. Phytomedicine.

[127] Nepali K., Sharma S., Ojha R., Dhar K. (2013). Vasicine and structurally related quinazolines. Med. Chem. Res..

[128] James P.A., Oparil S., Carter B.L., Cushman W.C., Dennison-Himmelfarb C., Handler J., Lackland D.T., LeFevre M.L., MacKenzie T.D., Ogedegbe O. (2014). Evidence-based guideline for the management of high blood pressure in adults: Report from the panel members appointed to the Eighth Joint National Committee (JNC 8). JAMA.

[129] Huang Y.L., Cui S.Y., Cui X.Y., Cao Q., Ding H., Song J.Z., Hu X., Ye H., Yu B., Sheng Z.F. (2016). Tetrandrine, an alkaloid from *S. tetrandra* exhibits anti-hypertensive and sleep-enhancing effects in SHR via different mechanisms. Phytomedicine.

[130] Fossati E., Narcross L., Ekins A., Falgueyret J.P., Martin V.J. (2015). Synthesis of morphinan alkaloids in *Saccharomyces cerevisiae*. PLoS ONE.

[131] Hou Q., He W.J., Wu Y.S., Hao H.J., Xie X.Y., Fu X.B. (2020). Berberine: A traditional natural product with novel biological activities. Altern. Ther. Health Med..

[132] Gorgani L., Mohammadi M., Najafpour G.D., Nikzad M. (2017). Piperine—The bioactive compound of black pepper: From isolation to medicinal formulations. Compr. Rev. Food Sci. F.

[133] Rozengart E.V., Basova N.E. (2001). Ammonium compounds with localized and delocalized charge as reversible inhibitors of cholinesterases of different origin. J. Evol. Biochem. Physiol..

[134] Sun J.Y., Zhu M.Z., Wang S.W., Miao S., Xie Y.H., Wang J.B. (2007). Inhibition of the growth of human gastric carcinoma *in vivo* and *in vitro* by swainsonine. Phytomedicine.

[135] Martínez-Pinilla E., Oñatibia-Astibia A., Franco R. (2015). The relevance of theobromine for the beneficial effects of cocoa consumption. Front. Pharmacol..

[136] Burchfield G., Hopes M. (1997). What’s your Poison: Caffeine.

[137] Schraufnagel D.E., Blasi F., Drummond M.B., Lam D.C., Latif E., Rosen M.J., Sansores R., Van Zyl-Smit R. (2014). Electronic cigarettes. A position statement of the forum of international respiratory societies. Am. J. Respir. Crit. Care Med..

[138] Sajja R.K., Rahman S., Cucullo L. (2016). Drugs of abuse and blood-brain barrier endothelial dysfunction: A focus on the role of oxidative stress. J. Cereb. Blood Flow Metab..

[139] Zhang X.-Y., Bi R.-Y., Zhang P., Gan Y.-H. (2018). Veratridine modifies the gating of human voltage-gated sodium channel Nav1.7. Acta Pharmacol. Sin..

[140] Povšnar M., Koželj G., Kreft S., Lumpert M. (2017). Rare tradition of the folk medicinal use of *Aconitum spp*. is kept alive in Solčavsko, Slovenia. J. Ethnobiol. Ethnomed..

[141] O’Brien P., Carrasco-Pozo C., Speisky H. (2006). Boldine and its antioxidant or health-promoting properties. Chem. Biol. Interact..

[142] Zheng J., Zheng S., Feng Q., Zhang Q., Xiao X. (2017). Dietary capsaicin and its anti-obesity potency: From mechanism to clinical implications. Biosci. Rep..

[143] Friedman M. (2006). Potato glycoalkaloids and metabolites:  roles in the plant and in the diet. J. Agric. Food Chem..

[144] Friedman M. (2002). Tomato glycoalkaloids: Role in the plant and in the diet. J. Agric. Food Chem..

[145] Li Z.C., Kong X.B., Mai W.P., Sun G.C., Zhao S.Z. (2015). Synthesis and antimicrobial activity of 9-*O*-substituted palmatine derivatives. Indian J. Pharm. Sci..

[146] Achan J., Talisuna A.O., Erhart A., Yeka A., Tibenderana J.K., Baliraine F.N., Rosenthal P.J., D’Alessandro U. (2011). Quinine, an old anti-malarial drug in a modern world: Role in the treatment of malaria. Malar. J..

[147] Walker N., Howe C., Glover M., McRobbie H., Barnes J., Nosa V., Parag V., Bassett B., Bullen C. (2014). Cytisine versus nicotine for smoking cessation. N. Engl. J. Med..

[148] Pomara C., Cassano T., D’Errico S., Bello S., Romano A.D., Riezzo I., Serviddio G. (2012). Data available on the extent of cocaine use and dependence: Biochemistry, pharmacologic effects and global burden of disease of cocaine abusers. Curr. Med. Chem..

[149] Haller C.A., Jacob P., Benowitz N.L. (2002). Pharmacology of ephedra alkaloids and caffeine after single-dose dietary supplement use. Clin. Pharmacol. Ther..

[150] Khansari M., Sohrabi M., Zamani F. (2013). The useage of opioids and their adverse effects in gastrointestinal practice: A review. Middle East J. Dig. Dis..

[151] Parthvi R., Agrawal A., Khanijo S., Tsegaye A., Talwar A. (2019). Acute opiate overdose: An update on management strategies in emergency department and critical care unit. Am. J. Ther..

[152] Pérez E.G., Cassels B.K., Cordell G.A. (2010). Alkaloids from the Genus *Duguetia*. The Alkaloids: Chemistry and Biology.

[153] Maheshwari N.O., Khan A., Chopade B.A. (2013). Rediscovering the medicinal properties of *Datura sp*.: A review. J. Med. Plants Res..

[154] Rick C.M., Uhlig J.W., Jones A.D. (1994). High R-tomatine content in ripe fruit of Andean *Lycopersicon esculentum* Var. cerasiforme: Developmental and genetic aspects. Proc. Natl. Acad. Sci. USA.

[155] Heal K.G., Taylor-Robinson A.W. (2010). Tomatine adjuvantation of protective immunity to a major pre-erythrocytic vaccine candidate of malaria is mediated *via* CD8+ T cell release of IFN-γ. J. Biomed. Biotechnol..

[156] Zhang N., Lian Z., Peng X., Li Z., Zhu H. (2017). Applications of higenamine in pharmacology and medicine. J. Ethnopharmacol..

[157] Morikawa T., Kitagawa N., Tanabe G., Ninomiya K., Okugawa S., Motai C., Kamei I., Yoshikawa M., Lee I.-J., Muraoka O. (2016). Quantitative determination of alkaloids in lotus flower (flower buds of *Nelumbo nucifera*) and their melanogenesis inhibitory activity. Molecules.

[158] Ka S.M., Kuo Y.C., Ho P.J., Tsai P.Y., Hsu Y.J., Tsai W.J., Lin Y.L., Shen C.C., Chen A. (2010). (S)-armepavine from Chinese medicine improves experimental autoimmune crescentic glomerulonephritis. Rheumatology.

[159] Chen C., Lin L., Xiao J., Sarker S., Asakawa Y. (2020). Alkaloids in diet. Handbook of Dietary Phytochemicals.

[160] Winzer T., Gazda V., He Z., Kaminski F., Kern M., Larson T.R., Li Y., Meade F., Teodor R., Vaistij F.E. (2012). A *Papaver somniferum* 10-gene cluster for synthesis of the anticancer alkaloid noscapine. Science.

[161] Bhambhani S., Kondhare K.R., Giri A.P. (2021). Advanced genome editing strategies for manipulation of plant specialized metabolites pertaining to biofortification. Phytochem. Rev..

